# MMP 9-instructed assembly of bFGF nanofibers in ischemic myocardium to promote heart repair

**DOI:** 10.7150/thno.77345

**Published:** 2022-10-17

**Authors:** Yaguang Wang, Di Wang, Chao Wu, Bin Wang, Shufang He, Hua Wang, Gaolin Liang, Ye Zhang

**Affiliations:** 1 Department of Anesthesiology, The Second Affiliated Hospital of Anhui Medical University, 678 Furong Road, Hefei 230601, PR China.; 2 Key Laboratory of Anesthesiology and Perioperative Medicine of Anhui Higher Education Institutes, Anhui Medical University, 678 Furong Road, Hefei 230601, PR China.; 3 Inflammation and Immune Mediated Diseases Laboratory of Anhui Province, Anhui Medical University, 81 Meishan Road, Hefei 230032, PR China.; 4 State Key Laboratory of Bioelectronics School of Biological Sciences and Medical Engineering Southeast University, 2 Sipailou Road, Nanjing 210096, PR China.

**Keywords:** Assembly, Basic fibroblast growth factor, Matrix metallopeptidase 9, Myocardial ischemia-reperfusion, Slow-release

## Abstract

**Background**: The only effective treatment for myocardial infarction (MI) is the timely restoration of coronary blood flow in the infarcted area, but further reperfusion exacerbates myocardial injury and leads to distal coronary no-reflow, which affects patient prognosis. Angiogenesis could be an important therapeutic strategy for re-establishing the blood supply to save the ischemic myocardium after MI. Basic fibroblast growth factor (**bFGF**) has been shown to promote angiogenesis. However, direct intravenous administration of **bFGF** is not a viable option given its poor half-life *in vivo*.

**Methods**: Herein, we developed a peptide Lys-Lys-Pro-Leu-Gly-Leu-Ala-Gly-Phe-Phe (**K2**) to encapsulate **bFGF** to form **bFGF@K2 micelle** and proposed an enzyme-instructed self-assembly (EISA) strategy to deliver and slowly release **bFGF** in the ischemic myocardium.

**Results**: The **bFGF@K2 micelle** exerted a stronger cardioprotective effect than free **bFGF** in a rat model of myocardial ischemia-reperfusion (MI/R). *In vitro* results revealed that the **bFGF@K2 micelle** could be cleaved by matrix metallopeptidase 9 (MMP-9) to yield **bFGF@Nanofiber** through amphipathic changes. *In vivo* experiments indicated that intravenous administration of **bFGF@K2 micelle** could lead to their restructuring into **bFGF@Nanofiber** and long term retention of **bFGF** in the ischemic myocardium of rat due to high expression of MMP-9 and assembly-induced retention (AIR) effect, respectively. Twenty-eight days after MI/R model establishment, **bFGF@K2 micelle** treatment significantly reduced fibrosis and improved cardiac function of the rats.

**Conclusion:** We predict that our strategy could be applied in clinic for MI treatment in the future.

## Introduction

Myocardial infarction (MI) is often accompanied by adverse cardiac events, such as arrhythmia, enlarged infarct size, and persistent ventricular systolic dysfunction 1. To treat MI, it is important to restore blood supply to the ischemic myocardium in a timely manner to prevent tissue necrosis 2. However, reperfusion of blood flow to the ischemic myocardium may exacerbate cellular injury, which is known as myocardial ischemia-reperfusion (MI/R) injury 3. This MI/R injury is mainly attributed to endothelial swelling, myocardial edema, and the inflammatory response, and it may result in distal coronary no-reflow 4. Recent advances in percutaneous intervention, drug therapy, and artery bypass grafts have led to a significant reduction in the mortality rate of patients associated with MI. Nevertheless, even after timely reperfusion, many patients still develop progressive heart failure due to ventricular remodeling-related complications, which seriously impaired the prognosis and life quality of the patients 5, 6. Therefore, how to enhance cardioprotection (e.g., through angiogenesis) and reduce cardiomyocyte death after MI are important for MI treatment but remain challenging.

Current strategies to promote angiogenesis of ischemic myocardial tissues mainly use therapeutic agents, such as strontium ions 7, growth factors 8, 9, and siRNA-21 10. And these agents have shown promising effects on myocardial remodeling alleviation and heart function recovery enhancement. In particular, members of the fibroblast growth factor (FGF) family have a strong capacity to promote angiogenesis and are frequently used to repair tissue damage 11. Among them, basic fibroblast growth factor (**bFGF**), a key member of the FGF family, plays a significant role in the treatment of various ischemic diseases and injuries, such as ischemic stroke 12, 13, wounds 14, and spinal cord injuries 15. Studies have shown that **bFGF** increases myocardial collateral blood flow in MI or MI/R animal models 16-18. However, although a single intracoronary infusion of **bFGF** was found to alleviate symptoms of angina in clinical trials, it failed to improve the exercise tolerance or myocardial perfusion 19. This deficiency may attribute to its poor half-life in blood circulation, rapid loss of biological activity under enzymatic degradation, or rapid diffusion from the site of administration 20, 21.

To further prolong drug retention, as a nanofibrous three-dimensional scaffolds, electrospun fibers exhibit promising drug delivery capabilities 22, 23. Moreover, laying electrospun fiber patches to the epicardium of the infarcted region can normally generate the greatest cardiac retention 24. However, this kind of treatment is difficult to implement because of large trauma range, and is not suitable for patients with MI. Recently, hydrogels have been used as the main carriers for their encapsulations. In preclinical studies 8, 25, 26 or clinical trials 27, 28, direct intramyocardial injections of the hydrogels at the site of ischemic myocardium significantly improved the retentions of the drug. Nevertheless, safety of the intramyocardially injected hydrogels needs further evaluation at the clinical level 27. This is because that, due to the weakened ischemic myocardial wall, intramyocardial injection increases the risk of heart rupture in the acute phase after MI/R 29, 30. Therefore, there is an ongoing demand to develop a “smart” intravenous drug administration method to slowly release drugs (e.g., **bFGF**) within the ischemic myocardium.

The *in situ* assembly-induced retention (AIR) effect of supramolecular peptide nanostructures has shown excellent drug-carrying capacity. These nanostructures can self-assemble within target organs and slowly release drugs to improve diagnosis and treatment efficiencies 31-34. There are many stimuli that can trigger the AIR effect, including reduced glutathione (GSH) 35, pH 32, and enzymes 33, 34, 36. Enzyme-instructed self-assembly (EISA), which enables more precise formation of *in situ* assemblies under disease-specific enzymes *in vivo*, has been widely used in biomaterial studies. In addition, peptides for EISA strategies self-assemble *via* noncovalent interactions and show high biocompatibility and degradability 37, 38, making them suitable candidates for organ protections. Recently, we proposed an EISA strategy for alkaline phosphatase (ALP)-triggered local self-assembly of dexamethasone (Dex), which significantly improves the anti-inflammatory effects of Dex for hepatic fibrosis treatment 36. Despite systemic delivery of supramolecular nanofibers approach has significantly prolonged drug retention within the ischemic myocardium 39, 40, a “smart” EISA strategy capable of assembling and slow releasing drug within the ischemic myocardium has not been reported.

**Figure 1:**
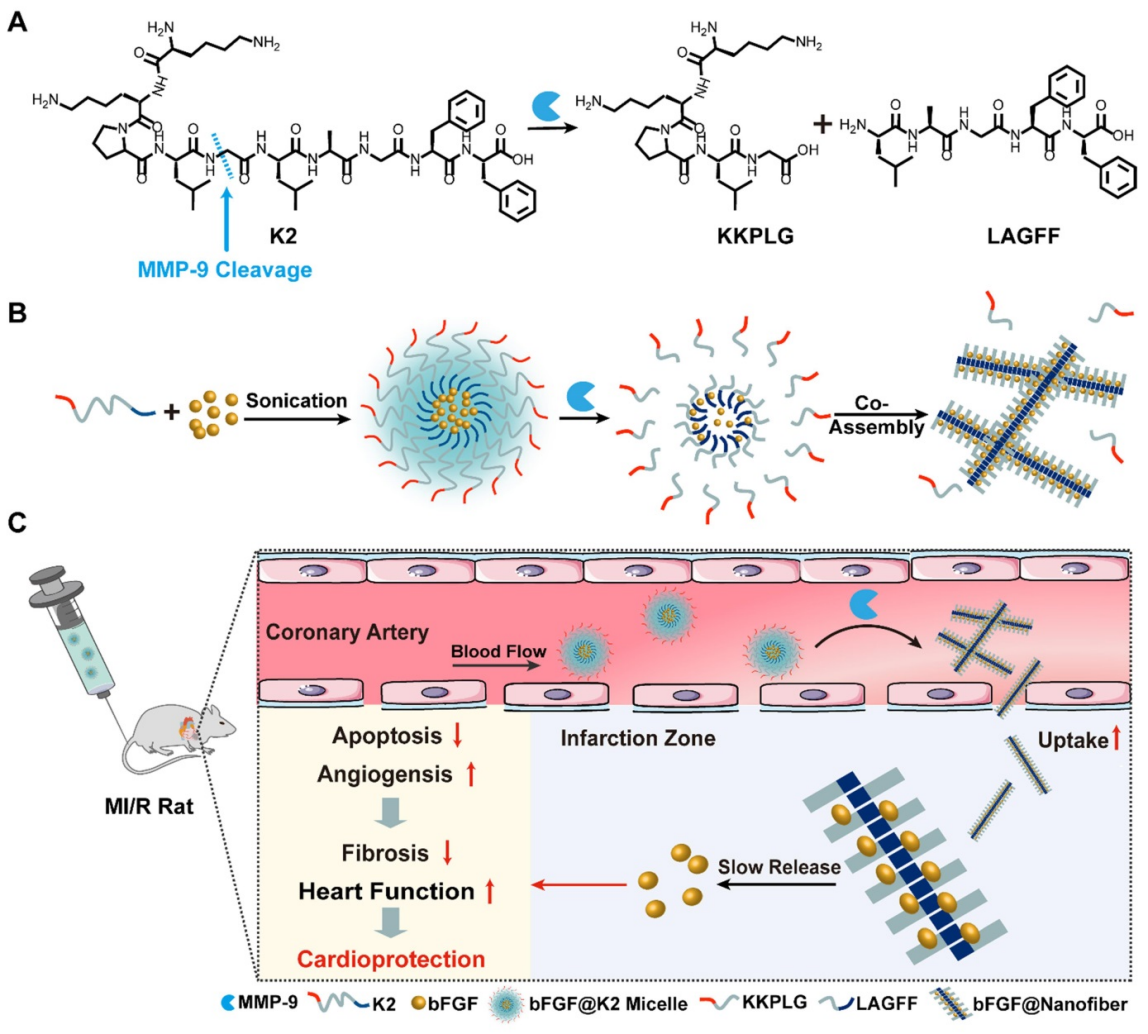
(A) Chemical structure of the MMP-9-responsive peptide **K2**. (B) Schematic illustration of the composition of **bFGF@K2 micelle** and its transformation to **bFGF@Nanofiber** upon MMP-9 cleavage. (C) Schematic illustration of the conversion of **bFGF@K2 micelle** to **bFGF@Nanofiber** in response to high expression of MMP-9 in the ischemic myocardium after intravenous injection. Slowly release of **bFGF** inhibited fibrosis and improved cardiac function in MI/R rats by inhibiting apoptosis and promoting angiogenesis in ischemic myocardium.

Matrix metallopeptidase 9 (MMP-9) expression is upregulated after MI. The expression of MMP-9 increases sharply within 24 h post MI 41 and remains high for 15-28 days 8, 42. Therefore, MMP-9 is a reasonable target enzyme in the treatment of MI/R. Moreover, the characteristic vascular leakage after MI leads to an enhanced permeability and retention (EPR) effect of ischemic myocardium which is beneficial for nanostructure accumulations 43. Based on these findings, we intended to design a **bFGF**-containing nanostructure which firstly would accumulate in ischemic myocardium. Then, *in situ* MMP would transform the nanostructure to form **bFGF** nanofibers in ischemic myocardium, rendering a slow release of **bFGF** to promote cardiac repair. Thus, we designed a supramolecular precursor peptide Lys-Lys-Pro-Leu-Gly-Leu-Ala-Gly-Phe-Phe (**K2**) which contains a Pro-Leu-Gly-Leu-Ala-Gly (PLGLAG) sequence for MMP-9 cleavage (PLG↓LAG) 44. Upon MMP cleavage, **K2** is converted to an amphiphilic product Leu-Ala-Gly-Phe-Phe (LAGFF) for nanofiber formation (Figure 1A). We found that, the peptide **K2** itself forms **K2 micelle** *in vitro* through its amphiphilic equilibrium, and the hydrophobic core of **K2 micelle** is able to load **bFGF** to form **bFGF@K2 micelle** (Figure 1B). In the presence of MMP, **K2** peptide on the surface of **bFGF@K2 micelle** is cleaved, transforming the micelles into** bFGF@Nanofiber**, as shown in Figure 1B. After the **bFGF@K2 micelle** are injected to the MI/R rats through tail vein, the micelles are circulated in ischemic myocardium *via* the bloodstream and on site accumulated due to the EPR effect of ischemic myocardium (Figure 1C). Then, an MMP-based EISA occurs, transforming the micelles into **bFGF@Nanofiber** nanofibers. EISA-formed nanofibers not only retain **bFGF** for a longer time but also slowly release it *via* the AIR effect (Figure 1C). Our approach will significantly improve the therapeutic efficacy of myocardial repair by promoting angiogenesis and inhibiting apoptosis in ischemic myocardium (Figure 1C).

## Results and Discussion

### Syntheses and characterization of bFGF@K2 micelle

We first synthesized and characterized the amphipathic peptide **K2** (Figure S1-3). The results of dynamic light scattering (DLS) showed that, at 1 mM **K2**, the **K2 micelle** had a mean particle size of 89.0 ± 15.8 nm and their sizes increased with the increase of **K2** concentration (Figure S4A-C). Zeta potential measurements showed that the zeta potential of **K2** was 13.9 ± 0.7 mV at pH 7.4 (Figure S5), and the isoelectric point (pI) value of recombinant **bFGF** was 9.6. Therefore, we thought that additional formation of **bFGF@K2 micelle** was through direct encapsulation of **bFGF** by the hydrophobic core of **K2 micelle** (Figure 1B). This type of core-shell structure of **bFGF@K2 micelle** well protects **bFGF** from enzymatic degradation during blood circulation. To investigate the encapsulation efficiency (EE%) of the **bFGF@K2 micelle**, a mixture of **bFGF** and **K2** was centrifuged after alternating vortexing and ultrasonication, and the concentration of **bFGF** in the supernatant was measured using enzyme-linked immunosorbent assay (ELISA). The EE% of the **bFGF@K2 micelle** is shown in Figure S6. As the concentration of **K2** increased, the maximum EE% reached 29.6 ± 6.9% at 1 mM **K2** (**bFGF** 1 μM), suggesting that the concentration of the peptide plays a pivotal role in EE%. The size of the **bFGF@K2 micelle** as measured by DLS was 227.1 ± 33.4 nm (Figure S4D), which met the particle size requirement of the EPR effect.

### MMP-9 triggered morphological transitions of peptide nanostructures for bFGF encapsulation

Next, we verified the *in vitro* conversion of micelles to nanofibers in response to MMP-9. First, MMP-9 (100 ng/mL) was added to a 1 mM **K2** solution at 37 °C (pH 7.4). The products of the reaction were characterized and quantified by high-performance liquid chromatography (HPLC). A peptide fragment LAGFF obtained by solid phase peptide synthesis (SPPS) was used as a control for above HPLC analysis. Figure S7 shows that, upon 24 h incubation, 77% of **K2** was cleaved by MMP to yield LAGFF (Figure S8). As we know, MMP-9 has a pI of 5.7 and is negatively charged at physiological condition (i.e., pH 7.4). Therefore, its efficiency in cleaving cationic peptides through electrostatic interactions is significantly higher than its efficiency in cleaving negatively charged peptides, and the specific cleavage site is P1↓P1′ (G↓L) 45. In this study, at pH 7.4, **K2** is positively charged which is beneficial to efficient MMP-9 cleavage through electrostatic interaction to yield LAGFF.

**Figure 2:**
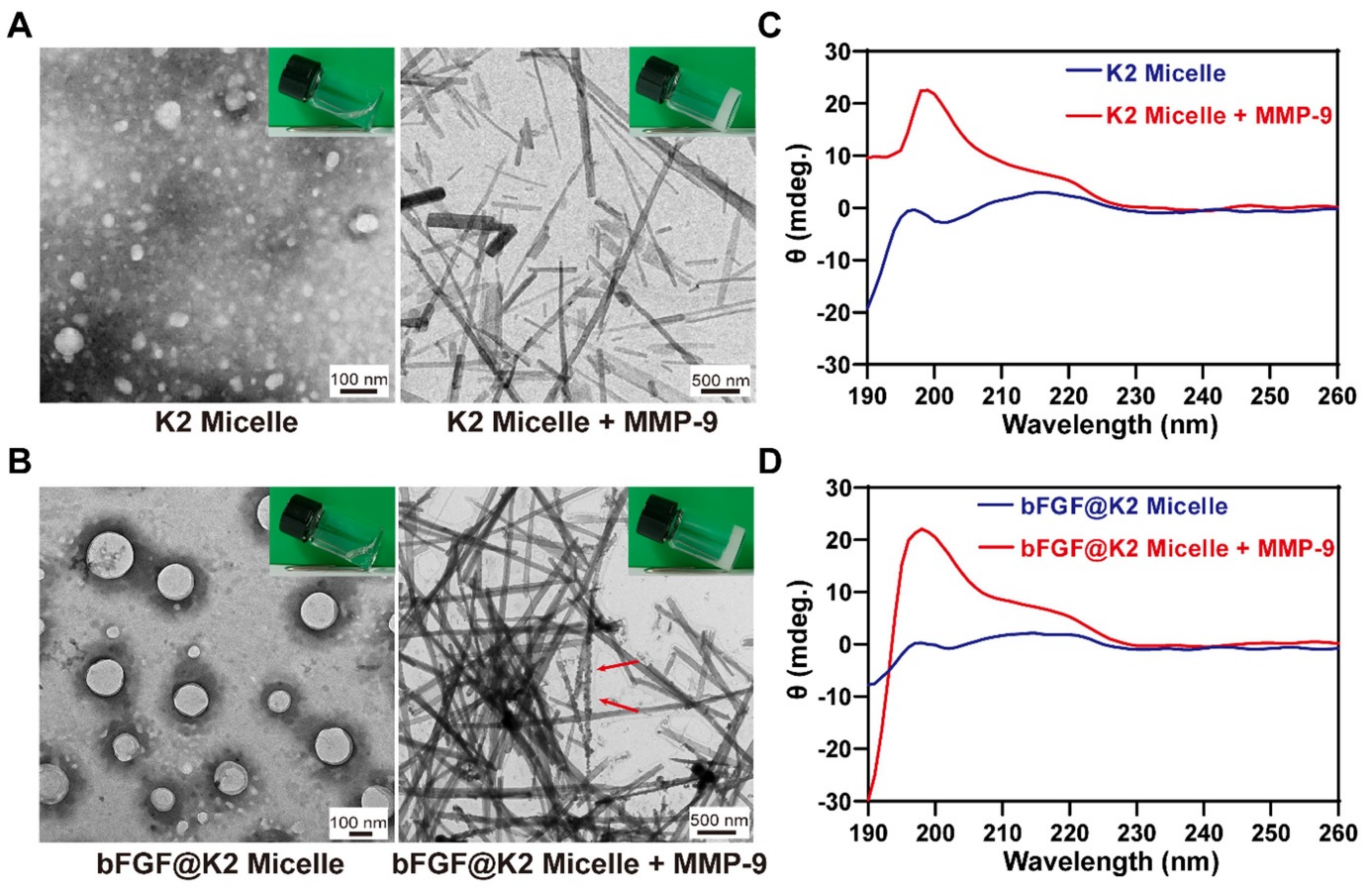
(A) TEM images of **K2 micelle** (left, inset is photograph of its solution) and 20 mM **K2 micelle** incubated with 2 μg/mL MMP-9 for 24 h (right, inset is photograph of the hydrogel). (B) TEM images of **bFGF@K2 micelle** (left, inset is photograph of its solution) and 20 mM **bFGF@K2 micelle** incubated with 2 μg/mL MMP-9 for 24 h (right, inset is photograph of the hydrogel). CD spectra of 20 mM** K2 micelle** (C) and 20 mM** bFGF@K2 micelle** (D) incubated with (or w/o) MMP-9 for 24 h.

Under transmission electron microscopy (TEM) observation, both **K2 micelle** and **bFGF@K2 micelle** showed uniform spherical structures (Figure 2A, B), whose sizes were in accordance with above DLS results, respectively. After **K2** were mixed with **bFGF**, spherical micellar structures were still maintained, indicating **bFGF** was effectively encapsulated into the hydrophobic core of **K2 micelle** to form **bFGF@K2 micelle**. Atomic force microscopy (AFM) images show that peptide **K2** sequences form spherical nanostructures (Figure S9A), and in the presence of **bFGF**, spherical aggregates are still observed (Figure S9B). After 24 h incubation of MMP-9 with **K2 micelle**, we found that the incubation solution transformed to opaque hydrogel (top right inset in Figure 2A). TEM image showed uniform LAGFF nanofibers in the enzymatic hydrogel with an average diameter of 59.0 ± 21.6 nm (right panel of Figure 2A), which were consistent with those in the synthetic LAGFF hydrogel obtained under a heating-cooling process (Figure S10). Similarly, we found that, after 24 h incubation with MMP, **bFGF@K2 micelle** transformed into opaque hydrogel (top right inset in Figure 2B). TEM image showed uniform nanofibers in the hydrogel with an average diameter of 85.7 ± 12.5 nm (right panel of Figure 2B). Interestingly, roundish particles of **bFGF** were found on the nanofibers (red arrows in Figure 2B), suggesting the enzymatic nanofibers of **bFGF@K2 micelle** were still able to load **bFGF**.

Next, the viscoelastic properties of above two hydrogels were investigated by rheology. First, dynamic strain sweeps were performed on the hydrogels. As shown in Figure S11, the storage modulus (G') values of these two hydrogels were higher than their loss modulus (G'') on strains ranging from 0.1% to 10%, implying that these two samples are hydrogels. Further, the strain amplitude was set at 0.1%, and the dynamic frequency sweeps of two hydrogels were tested. The G' and G'' values of these two hydrogels slightly increased with increase of frequency from 0.1 to 10 Hz, and the G' values were higher than their G'', indicating that these two hydrogels can tolerate external force. Meanwhile, the **bFGF@Nanofiber** hydrogel was elastically stronger than that hydrogel without** bFGF**, indicating that coassembly with **bFGF** protein can enhance the mechanical strength of hydrogels.

In a parallel study, circular dichroism (CD) spectra of the** K2 micelle** and **bFGF@K2 micelle** before and after MMP-9 treatment were obtained to investigate the secondary structure changes during their nanostructure transformations, respectively. As shown in Figure 2C,D, no secondary structure was formed in either **K2 micelle** or **bFGF@K2 micelle**. In the presence of MMP, besides the incubation solutions transformed into hydrogels as above mentioned, CD spectra of both **K2 micelle** and **bFGF@K2 micelle** incubations showed a positive peak near 198 nm, indicating β-sheet-like secondary structures were formed in the hydrogels (Figure 2C,D) 46. Above results showed that MMP-9 indeed triggered the transformation of micelles into nanofibers while in the meantime, did not affect the encapsulation of **bFGF** in the nanostructures.

To further determine the nanofiber formation ability of **bFGF@K2 micelles** under MMP-9 cleavage, we first examined the critical micelle concentration (CMC) of the synthetic fragment LAGFF. Gelation concentration-dependent transmittance measurements showed that the CMC of synthetic fragment LAGFF was 164.34 μM (Figure S12). Then, 100 ng/mL MMP-9 was incubated with **bFGF@K2 micelles** at different concentrations (0.25, 1.25, or 5 mM). From TEM images, we found that, with the decrease of **bFGF@K2 micelles** concentration, fiber number and diameter of **bFGF@Nanofibers** significantly decreased (Figure S13). Moreover, as shown in Figure S13, nanofibers were generated even at a micelle concentration of 0.25 mM. These indicate that the **bFGF@K2 micelles** can be cleaved by MMP-9 to form **bFGF@Nanofibers** at low concentrations.

### Assessment of bFGF@K2 micelles stability

Because MMP-9 is a secreted protease, elevated MMP-9 in the blood may have different effects on the stability of **bFGF@K2 micelles**. In order to evaluate the stability of **bFGF@K2 micelle** *in vivo*, we first detected the concentration of MMP-9 in blood and ischemic myocardium of MI/R rats using ELISA. As shown in Figure S14, MMP-9 in blood increased from 54.22 ± 19.45 ng/mL at 0 h to 196.47 ± 35.46 ng/mL at 36 h after MI/R injury, and MMP-9 in ischemic myocardium increased from 935.62 ± 125.27 ng/g at 0 h to 3048.37 ± 656.97 ng/g at 36 h. This illustrates that MMP-9 concentration in ischemic myocardium was much higher than that in the blood. Next, we extracted the serum of rats at 36 h post-MI/R, and then prepared the incubation solution with PBS at 1:1. After incubating the **bFGF@K2 micelles** with the above incubation solution for 2 h, 4 h, 8 h, or 16 h, respectively, the incubation solution was then centrifuged for 25 min at high speed. A stability study of **bFGF@K2 micelles** was conducted via TEM observation of the precipitation and measuring **bFGF** concentration in the supernatant with ELISA. As shown in Figure S15A, after 8 h of incubation, the micelles were still able to maintain their morphology, while a large number of nanofibers appeared after 16 h incubation. Likewise, no apparent free **bFGF** appeared in the supernatant within 8 h of incubation (Figure S15B). Above results indicated that the **bFGF@K2 micelles** had certain stability, which is appropriate for subsequent *in vivo* experiments.

### Cumulative release of bFGF from the bFGF@Nanofibers *in vitro*

After **bFGF@K2 micelles** were cleaved by MMP-9 to yield **bFGF@Nanofiber** hydrogels, we analyzed the release profile of **bFGF** from the **bFGF@Nanofibers** hydrogels *in vitro*. First, we incubated 500 µL PBS (0.01 M, pH 7.4) with 200 μL **bFGF@Nanofiber** hydrogel at 37 **°**C. At different time points (1, 2, 4, 8, or 14 d), 500 µL PBS was collected and replaced. The amount of **bFGF** in the collected PBS was measured using ELISA kits. As shown in Figure S16, constant and sustained release of **bFGF** was observed up to 14 d. In the first two days, the cumulative release of **bFGF** quickly reached 44.16 ± 5.85%. The cumulative release rates of **bFGF** increased with time and approached their plateaus at 8 d. At 14 d, cumulative release rate of **bFGF** was 72.32 ± 10.57%. The results of this experiment suggest that **bFGF** was released from **bFGF@Nanofibers** in a sustainable manner, which is suitable for *in vivo* experiments.

### Retention of bFGF@K2 micelle in the ischemic myocarium after intravenous injection

**Figure 3:**
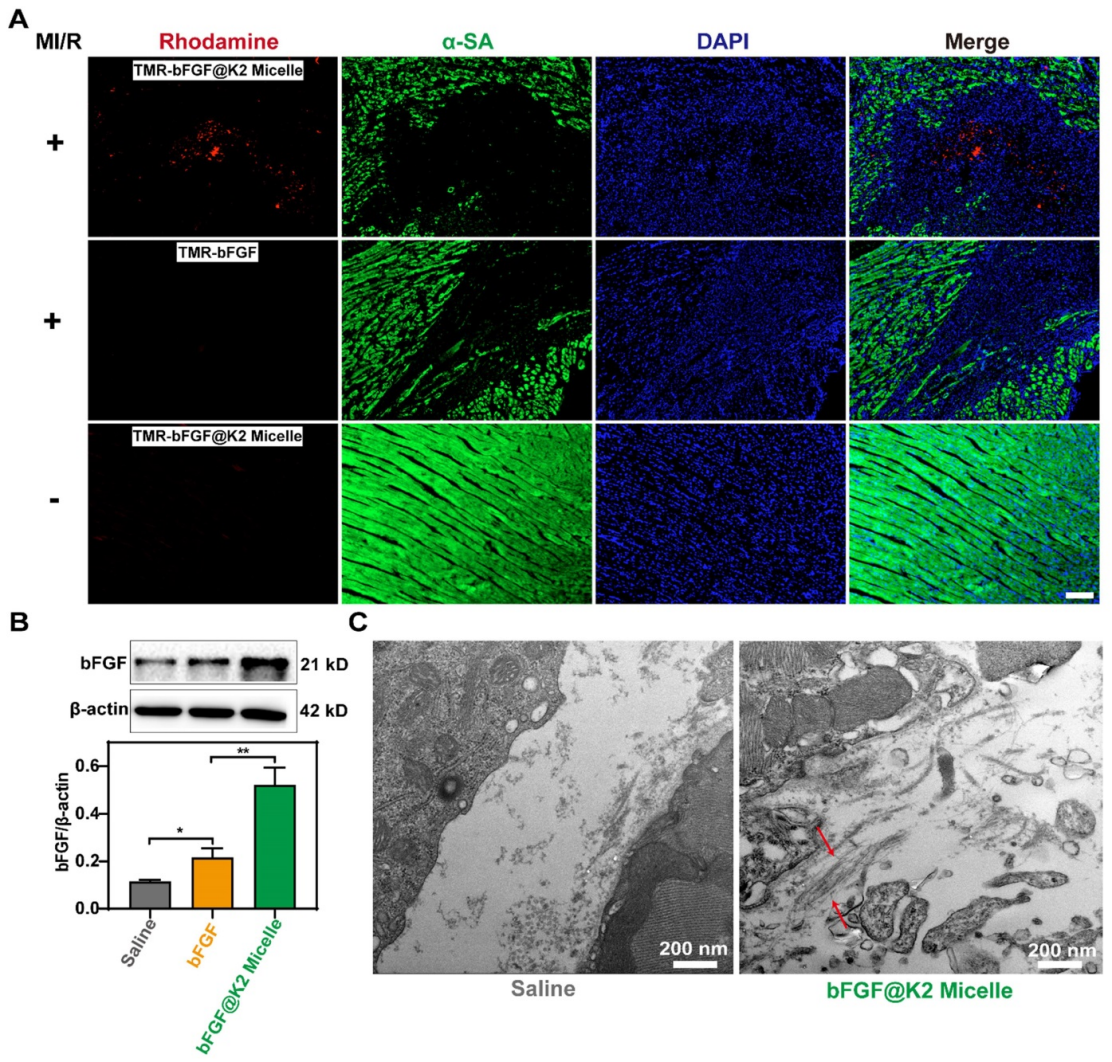
Accumulation and retention of **TMR-bFGF** in ischemic myocardium of MI/R rats 24 h after tail vein injection (n = 3). (A) Fluorescent images of myocardial tissues of MI/R rats (top lane) or normal rats (bottom lane) 24 h post tail vein injection of **TMR-bFGF@K2 micelle**. MI/R rats injected with free **TMR-bFGF** solution was used as a control (middle lane). **bFGF** was labeled with **TMR** (red), the myocardium was labeled with α-SA (green) and the nuclei were stained with DAPI (blue). Scale bar: 100 μm. (B) Western blotting analysis of the **bFGF** levels in the sites of ischemic myocardium of rats 24 h post Saline, **bFGF**, or **bFGF@K2 micelle** injection (n = 3); the results are presented as the mean ± SD of **bFGF**; *P < 0.05, **P < 0.01. (C) TEM images of the nanofibers in ischemic myocardium of MI/R rats 24 h after tail vein injection of **bFGF@K2 micelle**. Saline-injected MI/R rats were used as control.

Since self-assembly may have some effect on cell viability 45, 3-(4,5-dimethylthiazol-2-yl)-2,5-diphenyltetrazolium bromide (MTT) assay was performed to evaluate the cytotoxicity of **K2** and LAGFF (Figure S17). At a high peptide concentration up to 40 μg/mL and incubation time up to 48 h, approximately 99% or 84% of H2C9 cells were survived in **K2** or LAGFF, respectively, suggesting excellent biocompatibility of these two peptides. Considering the strong green autofluorescence from hemoglobin in ischemic myocardium 48, 5(6)-Carboxy-tetramethylrhodamine N-succinimidyl ester (i.e., **TMR-NHS**) with red fluorescence emission was used to label **bFGF** to trace its distribution *in vivo*. A rat model 24 h post-myocardial ischemia-reperfusion (MI/R) was used for this study. As shown in Figure 3A, 24 h after the intravenous injection of **TMR-bFGF@K2 micelle** (60 μg/kg **bFGF**), strong red fluorescence was observed at the site of ischemic myocardium in MI/R rat while neglectable red fluorescence was observed in the free **TMR-bFGF** group (60 μg/kg** bFGF**). Moreover, the red fluorescence was mainly located in the ischemic area but not in the distal viable myocardium. These results indicated that **TMR-bFGF@K2 micelle** was efficiently delivered and retained in ischemic area due to the EPR effect in this area. Additionally, red fluorescence still could be observed in **TMR-bFGF@K2 micelle** group at 48 h post-injection, and it gradually decreased over time till 72 h (Figure S18). To validate that **bFGF@K2 micelle** is MI-targeting, we also *i.v.* injected **TMR-bFGF@K2 micelle** into healthy rats. Only very weak fluorescence in cardiac sections of healthy rats was observed (Figure 3A), suggesting **bFGF@K2 micelle** cannot be retained in myocardium of a healthy rat whose blood vessels are not leaky. Besides, we also assessed the biodistribution of **TMR-bFGF@K2 micelle** in satellite organs of MI/R rats. As shown in Figure S19, red fluorescence was observed primarily in liver and spleen but significantly lower in the kidney, suggesting the **bFGF** was cleared by the macrophages in liver and spleen, as expected.

To further confirm that **bFGF@K2 micelle** was retained in the ischemic myocardium of MI/R rat, 24 h post injection, ischemic myocardial tissues were subjected to western blotting to measure **bFGF** levels. As Figure 3B showed, **bFGF** levels in the **bFGF@K2 micelle** group were significantly higher than those in the free **bFGF** group. To investigate **bFGF@Nanofiber** formation in the pathological area, the ischemic tissue was sectioned and examined under bio-TEM. As expected, nanofiber structures were found in the ischemic myocardium of MI/R rat 24 h post **bFGF@K2 micelle** injection (Figure 3C, indicated by red arrows). Collectively, above results suggested that, the leaky blood vessels in ischemic myocardium well retained **bFGF@K2 micelle** through the EPR effect, and high expression of MMP-9 in ischemic myocardium efficiently transformed **bFGF@K2 micelle** into **bFGF@Nanofiber** leading to long retention of bFGF in the area through AIR effect.

In order to explore the biodistributions and metabolic pathways of K2 micelles in MI/R model rats following intravenous injection, we constructed **TMR-K2 micelles** that could be traced by an *in vivo* fluorescence imaging system. 4 h or 24 h after intravenously administrated with **TMR-K2 micelles** or **TMR**, the MI/R rats were autopsied to collect major organs for *ex vivo* fluorescent imaging. As shown in Figure S20, at 4 h post injection, the heart fluorescent signal in **TMR-K2 micelles** group was significantly higher than that in **TMR** group. At 24 h, the fluorescence intensity of all major organs decreased significantly compared with that at 4 h. Nevertheless, at 24 h, the fluorescence intensity in the heart of **TMR-K2 micelles** group was still significantly higher than that of the TMR group. This indicates that the retention time of **TMR-K2 micelles** in the MI/R heart was significantly longer than that of **TMR** 24 h after injection. Apart from that in hearts, the fluorescence was primarily detected in the livers and kidneys, suggesting that the degraded micelles were excreted through the major metabolic organs.

### bFGF@K2 micelles reduce cardiomyocyte apoptosis and inhibit MMP-9 expression

**Figure 4:**
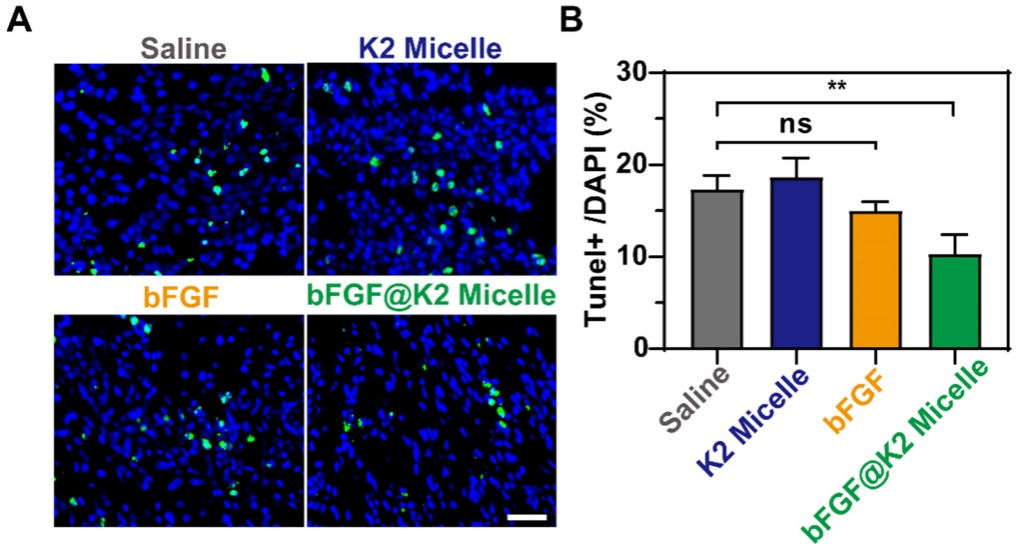
Representative images (A), and quantification (B) of TUNEL^+^ staining in the border zone of the ischemic myocardium of MI/R rats 2 days post tail vein injection of saline, **K2 micelle**,** bFGF**, or **bFGF@K2 micelle**. Scale bar: 50 μm (n = 3). The results are presented as the mean ± SD of TUNEL^+^ staining. **P < 0.01.

Inhibition of cardiomyocyte apoptosis can effectively attenuate myocardial tissue injury after MI/R 49, 50. Saline (200 μL), **K2 micelle** (14 mg/kg), **bFGF** solution (60 μg/kg), or **bFGF@K2 micelle** (60 μg/kg of **bFGF**) was intravenously injected into each MI/R model rat. Two days after injection, apoptotic cardiomyocytes were assessed by terminal deoxynucleotidyl transferase dUTP nick end labeling (TUNEL) staining. As shown in Figure 4, apoptotic rate of cardiomyocytes in the border area of the ischemic myocardium in **bFGF@K2 micelle** group was significantly lower than those in other three groups. Interesting, no significant difference in cardiomyocyte apoptosis was found among the saline, **bFGF**, and **K2 micelle** groups. These results indicated that **bFGF@K2 micelle** was able to slowly release **bFGF** via the AIR effect and therefore effectively inhibit cardiomyocyte apoptosis in ischemic myocardium of MI/R model rat.

Since the precursor peptide **K2** contains a PLGLAG sequence, which is the most commonly used MMP-9 substrate, it may inhibit the activity of MMP-9. Two days after injection, the levels of MMP-9 expression in the ischemic myocardial tissue of MI/R rat were measured using Western blotting. As Figure S21 shows, the MMP-9 levels in the **K2 micelle** and **bFGF@K2 micelle** groups were significantly lower than those in the saline and **bFGF** groups, suggesting MMP-9 was efficiently inhibited by the peptide **K2**. Nevertheless, at day 6 post injection, there was no significant difference of MMP-9 expression level among the four experimental groups. These results collectively indicated that **K2 micelle** could only inhibit MMP-9 activity in a short term (e.g., 2 days).

Since MMP-9 is mainly produced by leukocytes (neutrophils and macrophages) 51, and long duration of inflammation after MI/R sustains high expression level of MMP-9 for a long time 52, we thus repeatedly injected the micelles at day 2, day 5, and day 8 after MI/R in following experiments to ensure continuous inhibition on MMP-9 activity.

### bFGF@K2 micelle improved cardiac function

**Figure 5:**
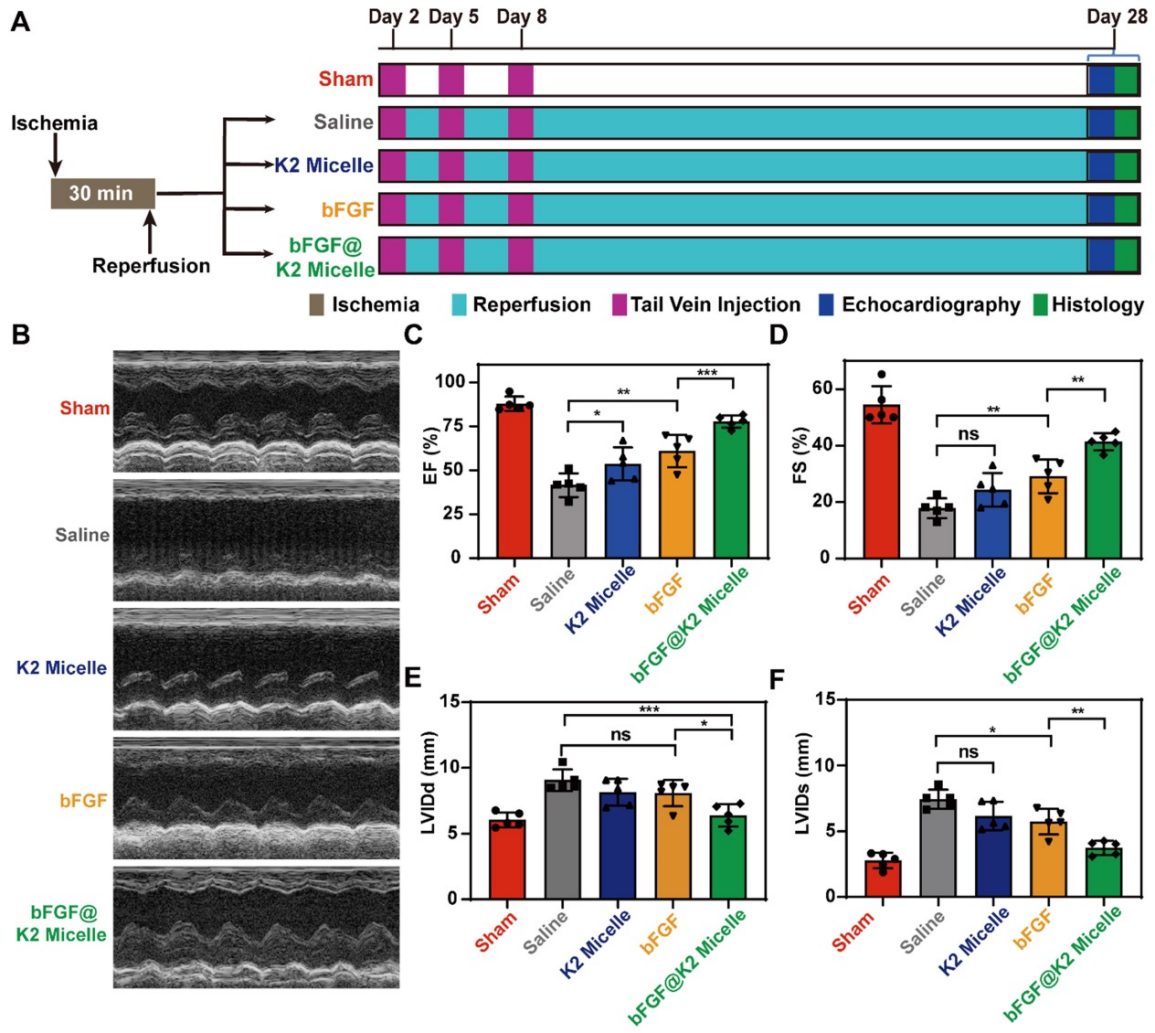
Noninvasive echocardiographic assessment of cardiac functions of the rats at day 28 post MI/R surgery and different treatments. (A) Schematic illustration of *in vivo* MI/R model establishment and the treatment process. (B) Representative echocardiograms obtained from the mid-papillary muscle region of the left ventricle of rats in each group (n = 5). Quantitative analyses of the EF value (C), FS value (D), LVIDd (E), and LVIDs (F) of rats in five groups. The results are presented as the mean ± SD of EF (%), FS (%), LVIDd (mm) and LVIDs (mm). *P < 0.05, **P < 0.01, and ***P < 0.001.

After retention and apoptosis evaluation of** bFGF@K2 micelle**, we tested the hypothesis that the AIR effect of **bFGF@K2 micelle** in the ischemic myocardium exerted cardioprotective effects in MI/R rats. We conducted following *in vivo* experiments according to a defined time schedule (Figure 5A). In detail, the rats were randomly divided into five groups (sham, saline, **K2 micelle**, **bFGF**, and **bFGF@K2 micelle**, n = 5 for each group). In sham group, the left anterior descending (LAD) of each rat was not ligated and saline was injected via tail vein. For rats in saline, **K2 micelle**, **bFGF**, or **bFGF@K2 micelle** group, their left anterior descending branches were ligated for 30 min and then reperfused for 24 h. Saline (200 μL), **K2 micelle** (14 mg/kg), **bFGF** solution (60 μg/kg), or **bFGF@K2 micelle** (60 μg/kg of bFGF) was intravenously injected into each MI/R model rat. Except those in sham group, body weights of the rats in four experimental groups declined in the first week after MI/R (Figure S22A). But there was no significant difference of the body weights among all fiver groups 28 days post MI/R surgery. Above results indicated successful establishment of the MI/R model (i.e., MI/R surgery brought acute but not long term side effects to the rats). As expected, the mortality of the rats in **bFGF@K2 micelle** group was lower than that of other three experimental groups that subjected to MI/R, though the difference was not statistically significant (Figure S22B).

As a noninvasive technology, echocardiography has been the preferred choice for cardiac function evaluation. The efficacy of *in vivo* cardiac repair of each group was evaluated by measuring echocardiograms of the rats at 28 days (Figure 5B). Compared to those in sham group, the rats in saline group showed serious cardiac dysfunction after MI/R as indicated by the markedly reduced left ventricular ejection fraction (EF) and fractional shortening (FS). However, treatment with **bFGF@K2 micelle** significantly improved the cardiac function of MI/R rats. By comparing with those in saline group, EF and FS values of **bFGF@K2 micelle** group were increased from 41.5 ± 6.7 % to 77.8 ± 3.5 %, and from 17.8 ± 3.5 % to 41.4 ± 3.0%, respectively (Figure 5C,D). In comparison, **bFGF** treatment exerted a less extent cardioprotective effects than **bFGF@K2 micelle** but still significantly increased both EF and FS values when compared with those in saline group. The K2 micelle also mildly increased the EF value while had few effects on the FS value (Figure 5C, D). In addition, **bFGF@K2 micelle** treatment also reduced the left ventricular internal diameter at end-diastole (LVIDd) and left ventricular internal diameter at end-systole (LVIDs) (Figure 5E,F). These results suggested that **bFGF@K2 micelle** exhibited stronger cardioprotective effect on MI/R rats than free **bFGF**. And this cardioprotective effect effectively prevented the cardiac function deterioration of the MI/R rats.

### bFGF@K2 micelle promoted angiogenesis in ischemic myocardium of the rats after MI/R

**Figure 6:**
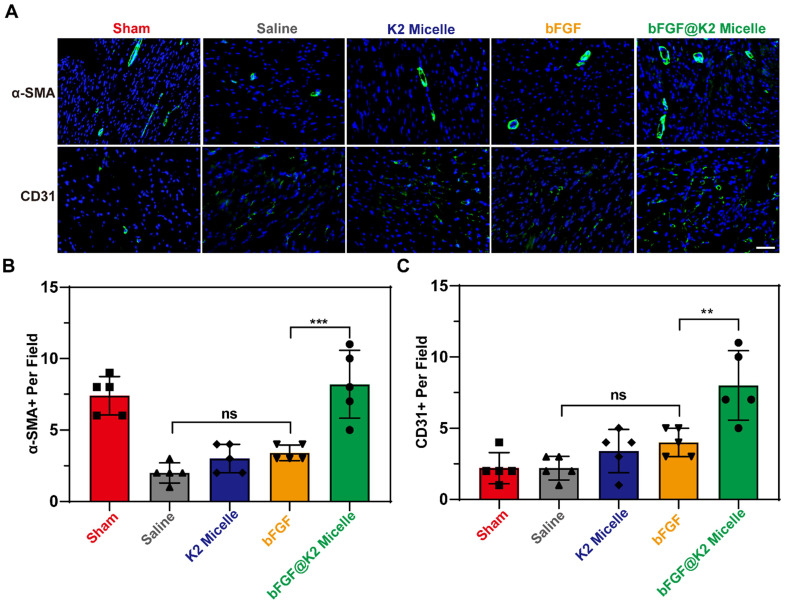
(A) Images of α-SMA and CD31 staining in Sham or MI/R heart sections of rats in each group 28 days post MI/R surgery (n = 5). Scale bar: 50 μm. Quantification of the α-SMA- positive (B), and CD31- positive (C) per field in heart sections from rats post MI/R. The results are presented as the mean ± SD of α-SMA^+^ and CD31^+^ staining. **P < 0.01 and ***P < 0.001.

As we know, **bFGF** has a strong ability to induce angiogenesis and can effectively promote neovascularization in ischemic myocardium after MI/R 16-18, 53. At day 28 post MI/R during which the rats received three doses of treatment (Figure 5A), we used anti-CD31 antibody to stain vascular endothelial and anti-α-SMA to stain the vascular smooth muscle for assessing angiogenesis degree in ischemic myocardium of the rats after MI/R. As Figure 6A,B showed, the number of α-SMA-positive per field in saline and **bFGF** groups were 2.00 ± 0.71 and 3.00 ± 1.00, respectively, while this number that in **bFGF@K2 micelle** group increased to 8.20 ± 2.39. Meanwhile, the number of CD31-positive per field was increased from 2.20 ± 0.84 in saline-treated group to 8.00 ± 2.45 in **bFGF@K2 micelle** group (Figure 6A, C). Again, the **K2 micelle** and** bFGF** had few effects than **bFGF@K2 micelle** on angiogenesis. These results indicated that **bFGF@K2 micelle** significantly improved the proangiogenic effect in ischemic in MI/R rats.

### bFGF@K2 micelle attenuated ventricular remodeling

**Figure 7:**
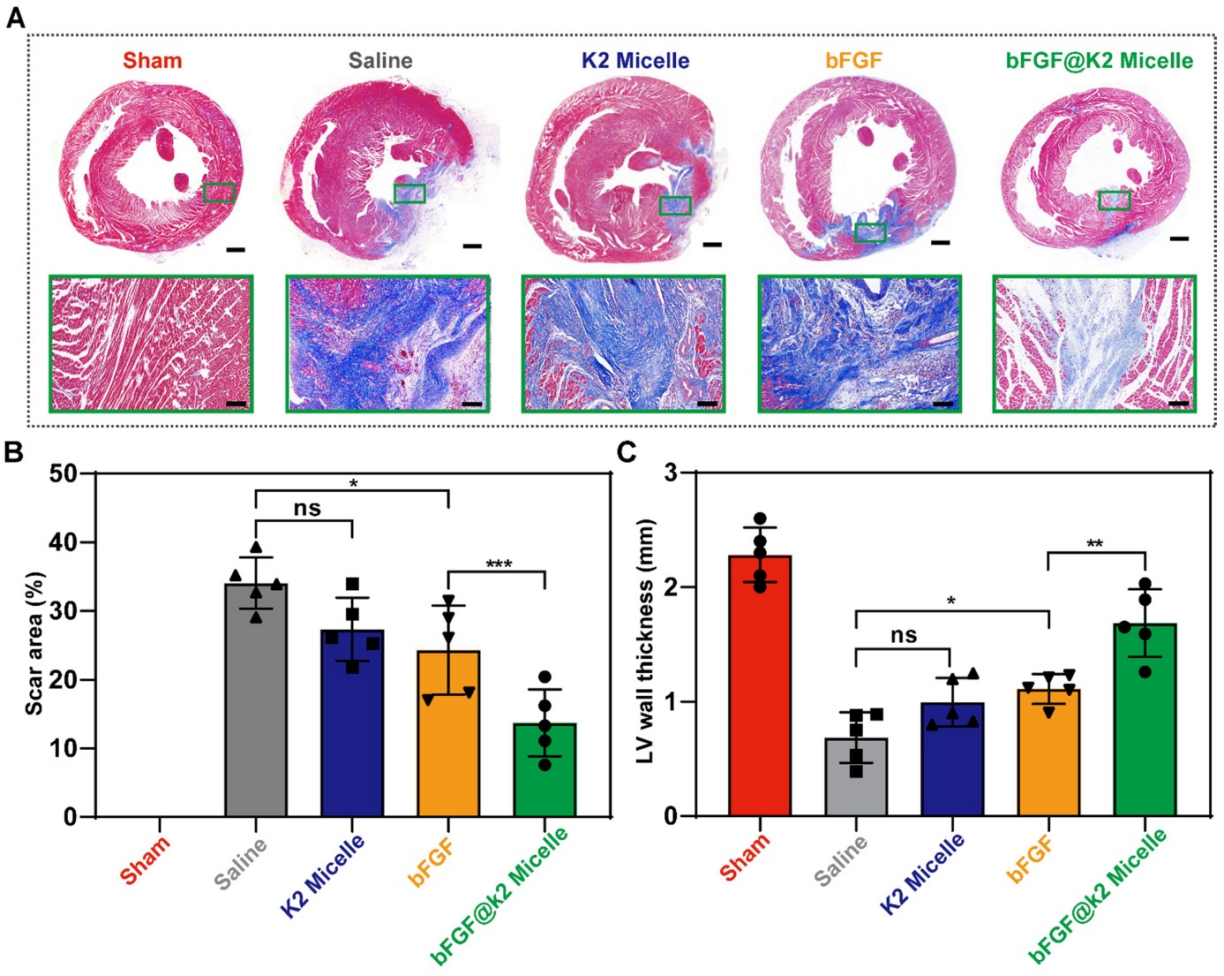
Masson's staining assessment of ventricular remodeling of rats at day 28 post MI/R surgery and different tratments. (A) Representative images of heart sections stained with Masson's staining of rats in five groups (n = 5). Quantitative analysis of the scar area (B), and left ventricular wall thickness (C) of the rats in each group. Scale bars: 1 mm for macro photos and 100 μm for micro photos. The results are presented as the mean ± SD of scar area (%) and left ventricular wall thickness (mm). *P < 0.05, and **P < 0.01.

The deterioration of cardiac function after MI/R often occurs due to left ventricular remodeling. Myocardial fibrosis and ventricular wall thickness are important parameters that are used to evaluate remodeling 54. Therefore, Masson's trichrome staining was performed to evaluate the degree of left ventricular remodeling 28 days after MI/R (Figure S23). The results showed that a mass of collagen fibers (stained blue) were deposited in the myocardium at 28 days after MI/R (Figure 7A). In addition, the left ventricular wall thickness in four experimental groups was reduced as compared to that in sham group. In consistent with the echocardiography results, **bFGF@K2 micelle** treatment significantly reduced collagen deposition and prevented the left ventricular wall thinning. In detail, as shown in Figure 7B,C, by comparing those in saline group, **bFGF@K2 micelle** treatment reduced the scar area from 34.0 ± 3.7% to 13.7 ± 4.9%, and increased the wall thickness from 0.69 ± 0.22 mm to 1.68 ± 0.30 mm. Although **bFGF** alone also reduced cardiac fibrosis and remodeling, its effects were significantly lower than those of **bFGF@K2 micelle**. In contrast, MI/R-induced cardiac fibrosis and remodeling were not improved by **K2 micelle** treatment.

### Biosafety evaluation of bFGF@K2 micelle

We also evaluated the biosafety of the **bFGF@K2 micelle** by hematoxylin-eosin staining (H&E) of the major organs of the MI/R rats after treatment. Except for the lung congestion caused by heart failure, other major organs of the MI/R rats after treatment did not show observable pathological change (Figure S24). The lung congestion in the rats in **bFGF@K2 micelle** group was significantly reduced compared with that in the MI/R rats of other three experimental groups (Figure S24, black box). These results indirectly indicated that **bFGF@K2 micelle** was nontoxic and also could significantly improve cardiac function.

## Conclusions

In conclusion, we rationally designed a biocompatible precursor peptide **K2** which is able to co-assemble with **bFGF** to form** bFGF@K2 micelle**. Due to the EPR effect of the leaky blood vessels in ischemic myocardium, **bFGF@K2 micelle** is circulated and accumulated in ischemic myocardium of MI/R rat. Then, a MMP-based EISA occurs, transforming the micelles into **bFGF@Nanofiber** nanofibers and slowly releasing **bFGF** *via* the AIR effect to enhance cardiac repair of the MI/R rats. *In vitro* experimental results showed that the **bFGF**@**K2 micelle** was hydrolyzed by MMP-9, transforming the micellar structure into **bFGF@Nanofiber**. *In vivo* experiments showed that **bFGF**@**K2 micelle** significantly increased the retention of **bFGF** through the formation of **bFGF@Nanofiber** within the ischemic myocardium of MI/R rats. Further *in vivo* studies showed that **bFGF**@**K2 micelle** had higher proangiogenic properties than free **bFGF**. Among four experimental groups, **bFGF**@**K2 micelle** showed the strongest inhibitory effects on cardiomyocyte apoptosis, myocardial fibrosis, and remodeling of the MI/R model rats. Moreover, the **bFGF@K2 micelle** exerted the best protective effects on cardiac function of the MI/R rats. Employing the EPR effect of ischemic myocardium and MMP 9-based EISA strategy, this work “smartly” transformed **bFGF@K2 micelle** into **bFGF@Nanofiber** on site, slowly releasing **bFGF** for effective promote heart repair of MI/R rats. We anticipate our smart strategy, as well as the biocompatible micelle, would be applied in clinic in near future.

## Materials and Methods

### Preparation and analysis of micelle

The **K2 micelle** forming peptide **K2** was dissolved in phosphate-buffered saline (PBS) at pH 7.4, and its self-assembly behavior was investigated after a cycle of alternating sonication and vortexing. Then, an appropriate amount of **K2**, combined with **bFGF**, was dissolved by sonication and vortexing, and the solvent was removed to form a transparent film, which was dissolved in PBS (pH 7.4) to prepare **bFGF@K2 micelle**. The dimensions of the micelles were determined by DLS measurements on a Malvern Zetasizer (Malvern, UK). The morphologies of **K2 micelle** and **bFGF@K2 micelle** pretreated with MMP-9 for 24 h were characterized by TEM (Thermo Fisher, USA) after negative staining with 1% phosphotungstic acid.

### Encapsulation efficiency of bFGF@K2 micelle

To determine the EE (%), the **bFGF@K2 micelle** was separated from the un-encapsulated **bFGF** containing supernatant by high-speed centrifugation at 16000 rpm for 30 min, and the supernatant was qualitatively analyzed by a **bFGF** ELISA kit (MultiSciences, China). The EE (%) was calculated by the following formula: EE (%) = (Initial **bFGF** concentration - concentration of supernatant **bFGF**)/Initial **bFGF** concentration × 100%.

### High-performance liquid chromatography

The cleaved peptide **K2** products were dissolved in 35% acetonitrile in water containing 0.1% TFA and purified through a preparatory C18RP column on an Agilent 1200.

### Echocardiography

Transthoracic echocardiography was performed with the echocardiography imaging system (Vinno 6, China). During the ultrasonography examination, the rat was anesthetized with isoflurane. The papillary muscles were used as a marker for short-axis imaging. The function and dimensions of the left ventricular were also recorded and measured at the same short axis with M-mode examination. LVDd and LVDs were recorded and measured using leading edge-to-leading edge technique. LV end-systolic volume (LVESV) and LV end-systolic volume (LVEDV) were estimated using Teichholz equations. EF% and FS% values were calculated as follows:

FS = [(LVDd-LVDs) /LVDd] × 100

EF = [(LVEDV-LVESV) /LVEDV]×100

## Supplementary Material

Supplementary figures and tables.
