# Supplementary material for: MMP 9-instructed assembly of bFGF nanofibers in ischemic myocardium to promote heart repair
Source: Theranostics. 2022 Oct 17;12(17):7237–49. doi: 10.7150/thno.77345 (PMC9691345; doi:10.7150/thno.77345)
Supplement: Supplementary file 1 — Supplementary figures and tables. [file thnov12p7237s1.pdf]

Supporting Information

MMP 9-instructed assembly of bFGF nanofibers in ischemic myocardium to promote heart repair

Yaguang Wang[1,2#], Di Wang[1,2#], Chao Wu[1,2#], Bin Wang[1,2], Shufang He[1,2], Hua Wang[3], Gaolin Liang[4*], Ye Zhang[1,2*]

1. Department of Anesthesiology, The Second Affiliated Hospital of Anhui Medical University, 678 Furong Road, Hefei 230601, PR China
2. Key Laboratory of Anesthesiology and Perioperative Medicine of Anhui Higher Education Institutes, Anhui Medical University, 678 Furong Road, Hefei 230601, PR China
3. Inflammation and Immune Mediated Diseases Laboratory of Anhui Province, Anhui Medical University, 81 Meishan Road, Hefei 230032, PR China
4. State Key Laboratory of Bioelectronics School of Biological Sciences and Medical Engineering Southeast University, 2 Sipailou Road, Nanjing 210096, PR China

[#]These three authors contributed equally to this work.

E-mail addresses: gliang@seu.edu.cn (G.-L. Liang), zhangy@ahmu.edu.cn (Y. Zhang).

Contents:

1. Methods

2. Solid-Phase peptide synthesis

3. Supporting figures and tables

1. Methods

Reagents and equipment

All chemicals are reagent grade and have not been further purified for use. 5(6)-Carboxy-tetramethylrhodamine N-succinimidyl ester (**TMR-NHS**) was purchased from Shanghai Yuanye Co.Ltd. Recombinant MMP-9 protein standard was purchased from Sino Biological (Beijing, China). Recombinant basic fibroblast growth factor (**bFGF**), MMP-9, alpha-smooth muscle actin (α-SMA) antibodies, and terminal deoxynucleotidyl transferase dUTP nick end labeling (TUNEL) staining kit were purchased from Beyotime (China). MMP-9 ELISA kit was purchased from MultiSciences (China). **bFGF** antibodies and MTT kit were purchased from Abcam (UK). CD31 and alpha-sarcomeric actin (α-SA) antibodies were purchased from Santa Cruz (USA). Electrospray ionization (ESI) mass spectra were obtained on a Finnigan LCQ (ThermoFisher Corporation). ^{1}H NMR and ^{13}C NMR spectra were obtained on a Bruker AV 400. The rheology test were obtained on MCR302 (Austria). Zeta and dynamic light scattering (DLS) were performed on a Malvern Nano-ZS ZEN3600 (UK). The circular dichroism (CD) spectra were obtained on J-1700 (JASCO, Japan).

Myocardial ischemia reperfusion animal model

Male Sprague Dawley rats aged 8-10 weeks, weighing 200-220 g, were obtained from the Experimental Animal Center of Anhui Medical University. All experimental procedures and protocols used in this study have been reviewed and approved by the Ethics Committee of Experimental Animals, Anhui Medical University (LLSC20190476).

The myocardial ischemia-reperfusion (MI/R) model was established via the left anterior descending branch ligated for 30 min and then reperfused. Briefly, all rats were anesthetized with

pentobarbital (40 mg/kg), then intubated and connected to a ventilator for mechanical ventilation, equipped with a Powerlab system to observe the electrocardiogram (ECG). The successful signs of ischemia were a pale ischemic appearance on the surface of the local myocardium, ST-segment elevation, and Q wave in lead II. The sham group underwent a sham operation without coronary artery ligation.

MTT assay

Cytotoxicity of peptide **K2** and LAGFF was evaluated using a 3-(4, 5-dimethylthiazol-2-yl)-2, 5-diphenyltetrazolium bromide (MTT) assay. Cells were seeded into 96-well cell culture plates at 5 × 10^4/well and incubated in a 5% CO_2 atmosphere at 37 ℃. The solutions of peptide diluted by DMEM (100 μL/well) at concentrations of 5, 10, 20, and 40 μg/mL in 100 μL medium were added to the wells, respectively. The cells were incubated with the peptides for 24 or 48 h. A solution of 5 mg/mL MTT dissolved in phosphate-buffered saline (PBS) (pH 7.4) (10 μL/well) was added to each well. A solution of DMSO (100 μL/well) was added to dissolve the formazan. The data were obtained using a 96-well plate reader to detect its absorption at 490 nm.

In vivo pharmacokinetic studies and tissue biodistribution

TMR-NHS was used to label the **bFGF**. 24h, 48h, and 72h after injection, hearts and major organs of rats were removed, fresh frozen in OCT, and cryosectioned. Heart cryosections were fixed with 4% paraformaldehyde in PBS and incubated with the antibody against α-SA to show the myocardium and then the **TMR** fluorescence was observed to identify the presence of **bFGF** in the ischemic myocardium. In addition, cryosections of liver, lung, spleen and kidney were directly used to observe the fluorescence of **TMR**. All pictures were captured using Zeiss microscopy and analyzed by NIH Image J software.

Western blot

The myocardial tissue including the ischemic area and border area was collected and lysates were prepared by freezing in radioimmunoprecipitation assay (RIPA) for 20 min. The cell debris was removed by centrifugation (12000 rpm, 4 ℃, 15 min), and then the cell lysates were mixed with SDS sample buffer at 100 ℃ for 10 min. Proteins were separated by sodium dodecyl sulfate-polyacrylamide gel electrophoresis (SDS-PAGE) and subjected to Immun-Blot polyvinylidene fluoride (PVDF) membrane (Bio-Rad). The membranes were blocked in 5% skim milk for 4 h, then incubated with primary antibody (anti-bFGF or anti-MMP-9), and the β-Actin and Gapdh were used as internal control, respectively. Membranes were washed and incubated with secondary antibodies conjugated to horseradish peroxidase. Immunoreactive bands were visualized with an ECL system. Protein bands were quantified by densitometric analysis using ImageJ software.

TUNEL staining

After treatment with saline, **K2 micelle**, **bFGF**, and **bFGF@K2 micelle** for 2 days, MI/R rats were sacrificed and the hearts were removed. Apoptotic cells in the border zone of the ischemic myocardium were analyzed using TUNEL kit according to the instructions, and DAPI counterstain was performed to visualize the nuclei. The TUNEL positive cells were observed under a fluorescent microscope and counted using NIH Image J software.

Immunohistochemistry and histopathology

After euthanasia, hearts of rats were taken out, fixed with 4% paraformaldehyde overnight at 4℃, then, fresh frozen in OCT and cryosectioned for immunohistochemistry. Blood vessels were stained with antibody against CD31 or α-SMA (1:500) mounted with fluoromount for fluorescent imaging under microscopy. For histopathological examination, hearts and other major organs were

formalin-fixed, paraffin-embedded, and sectioned. Masson's trichrome-staining was used to assess the fibrosis area and the left ventricular wall thickness of the heart slices. Sections of other major organs were stained with hematoxylin and eosin (H&E) to evaluate the biocompatibility of micelles using light microscopy. All images were analyzed using NIH Image J software.

2. Solid-Phase peptide synthesis

The peptide **K2** was synthesized with solid-phase peptide synthesis (SPPS), and protecting groups were removed with 95% TFA in CH_2Cl_2 for 3 h. Pure product **K2** was obtained after HPLC purification.

MS: calculated for compound **K2** [(M+H$^+$)]: 1077.6416; obsvd. HR-MS [(M+H$^+$)]: *m/z* 1077.6461 (Figure S1).

^{1}H NMR of **K2** (400 MHz, D_2O) δ 7.33-7.15 (m, 8 H), 7.13-7.03 (m, 2 H), 4.57 (dd, *J* = 8.7, 5.5 Hz, 2 H), 4.48 (dd, *J* = 8.4, 6.5 Hz, 1 H), 4.37 (dd, *J* = 8.3, 5.8 Hz, 1 H), 4.32 – 4.16 (m, 3 H), 3.97 (t, *J* = 6.6 Hz, 1 H), 3.90-3.67 (m, 5 H), 3.56 (dt, *J* = 10.0, 7.0 Hz, 1 H), 3.14 (dd, *J* = 14.0, 5.5 Hz, 1 H), 2.99-2.80 (m, 7 H), 2.21 (ddd, *J* = 15.0, 12.7, 7.1 Hz, 1 H), 1.95 (dq, *J* = 12.8, 6.5 Hz, 2 H), 1.83 (dq, *J* = 13.4, 6.5 Hz, 3 H), 1.73 (dt, *J* = 14.4, 7.7 Hz, 1 H), 1.71-1.47 (m, 11 H), 1.47-1.34 (m, 4 H), 1.31 (d, *J* = 7.2 Hz, 3 H), 0.82 (ddd, *J* = 19.7, 12.4, 6.2 Hz, 12 H) (Figure S2).

^{13}C NMR of **K2** (101 MHz, D_2O) δ 175.25, 175.23, 174.49, 174.15, 173.86, 172.36, 171.53, 171.36, 170.67, 169.51, 163.38, 163.02, 162.67, 162.31, 136.49, 136.29, 129.34, 129.17, 128.72, 128.70, 127.14, 120.69, 117.79, 114.89, 111.99, 60.21, 54.86, 53.94, 52.76, 52.67, 52.43, 51.81, 50.03, 48.06, 42.53, 42.36, 39.93, 39.84, 39.18, 39.00, 37.12, 36.75, 30.43, 29.92, 29.35, 26.46, 26.35, 24.70, 24.34, 24.28, 22.20, 22.11, 21.88, 21.20, 20.90, 20.81, 16.33 (Figure S3).

3. Supporting figures and tables

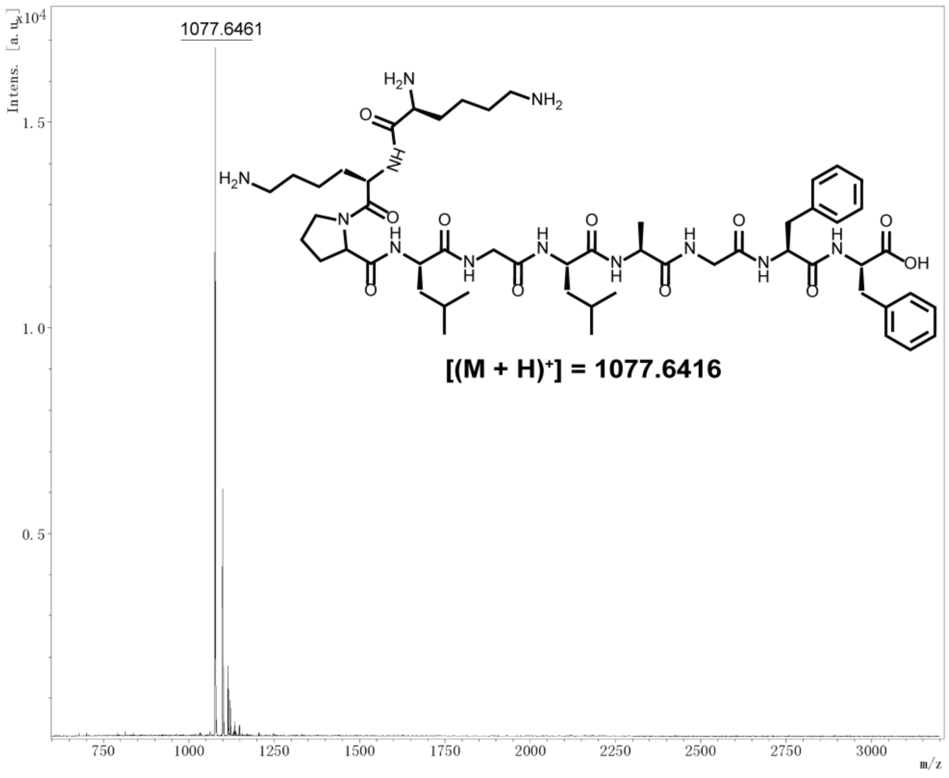

Figure S1. HR-MS spectrum of peptide **K2**.

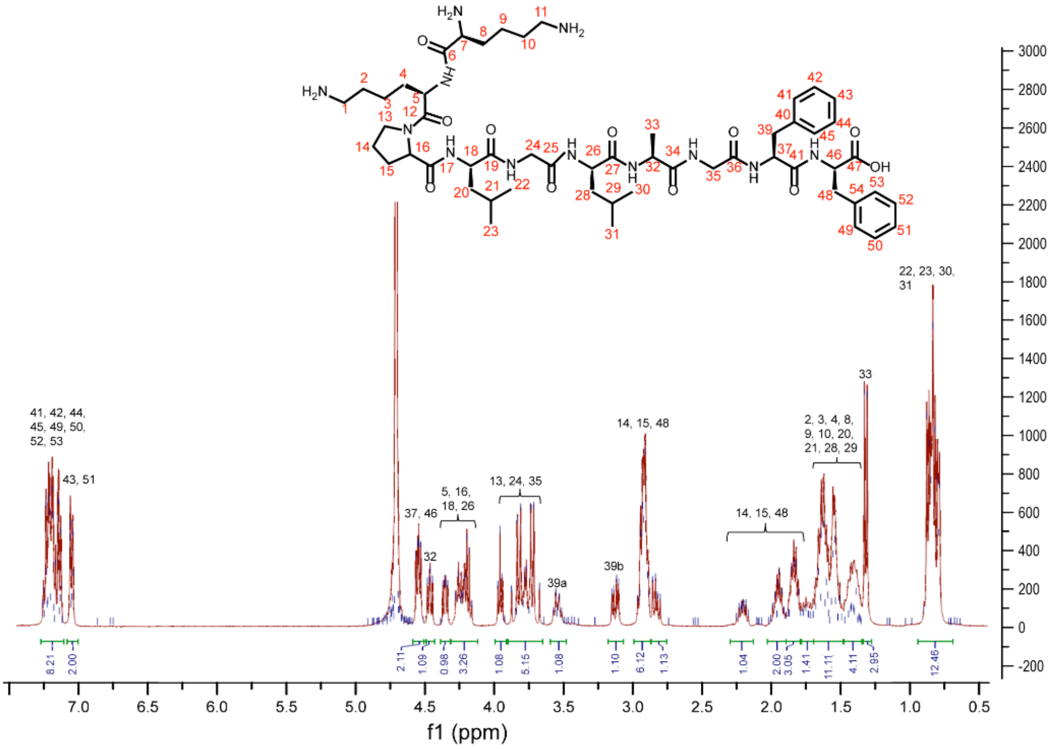

Figure S2. ¹H NMR spectrum of **K2** in D_2O.

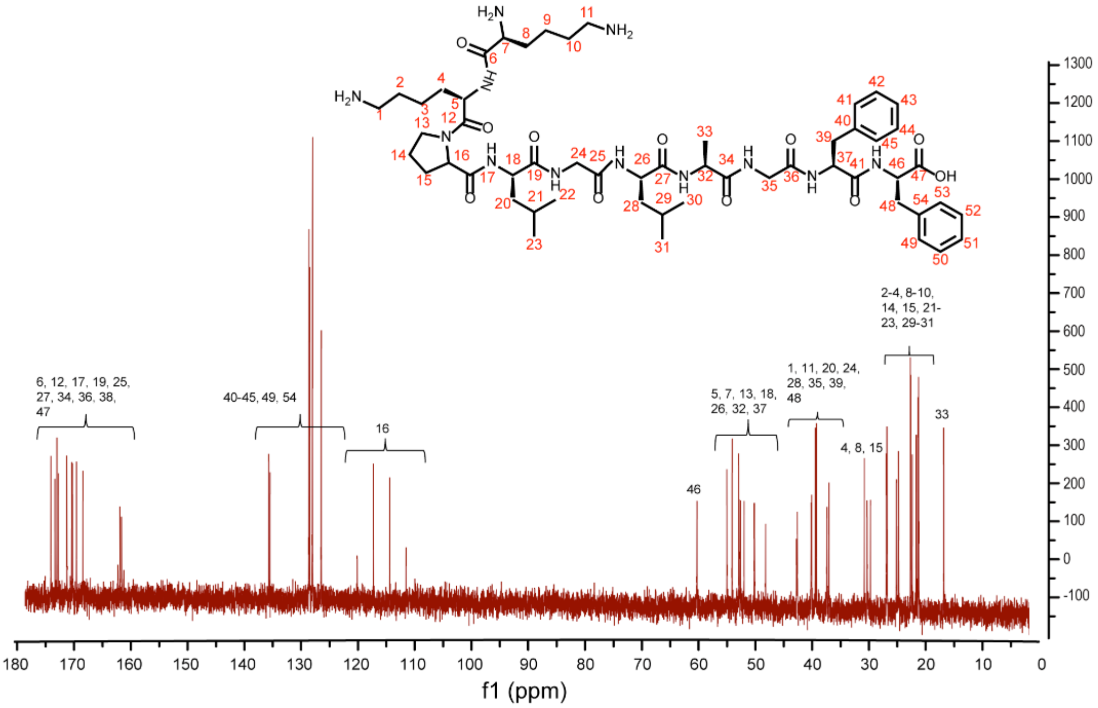

Figure S3. ^{13}C NMR spectrum of **K2** in D_2O.

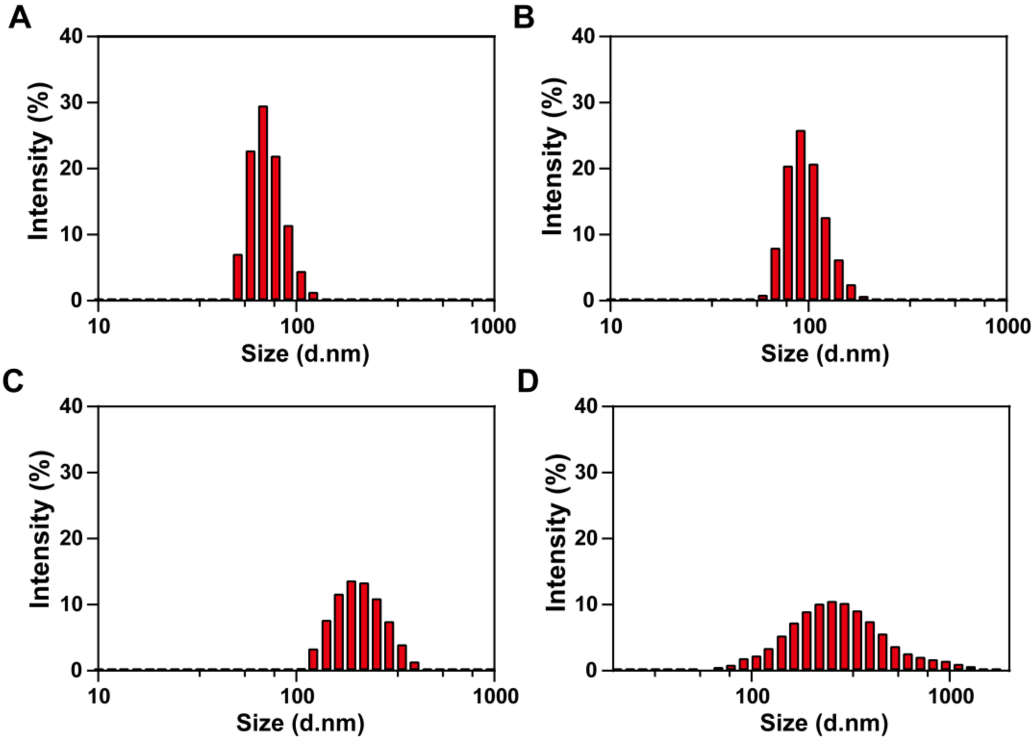

Figure S4. **K2 micelle** size under varying concentrations of peptide **K2**, (A) 0.5 mM, (B) 1 mM, (C) 2 mM and (D) **bFGF@K2 micelle** (1mM **K2**).

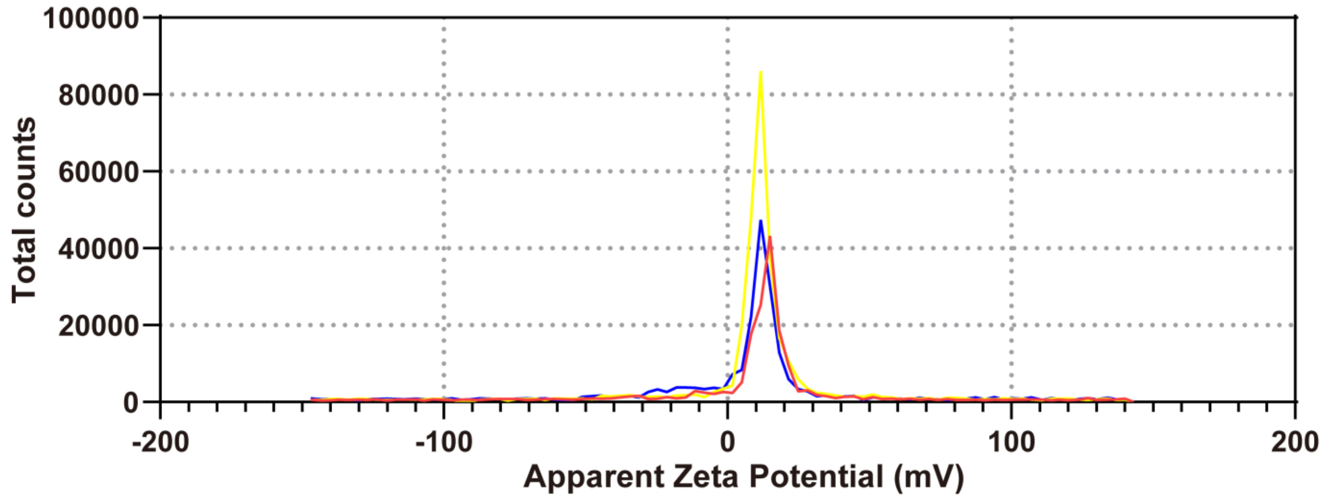

Figure S5. Triplicate measurements of the zeta potential of peptide **K2**.

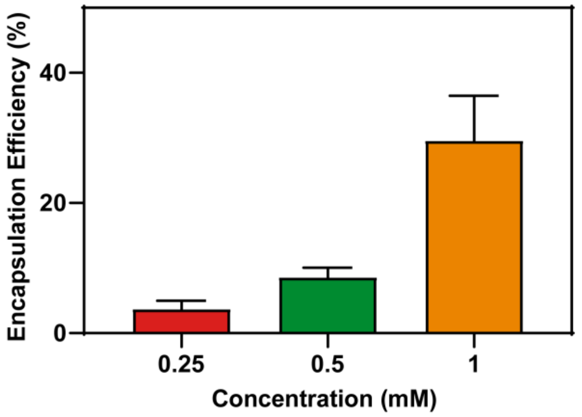

***Figure S6*.** The encapsulation efficiency of **bFGF@K2 micelle** at different peptide **K2** concentrations.

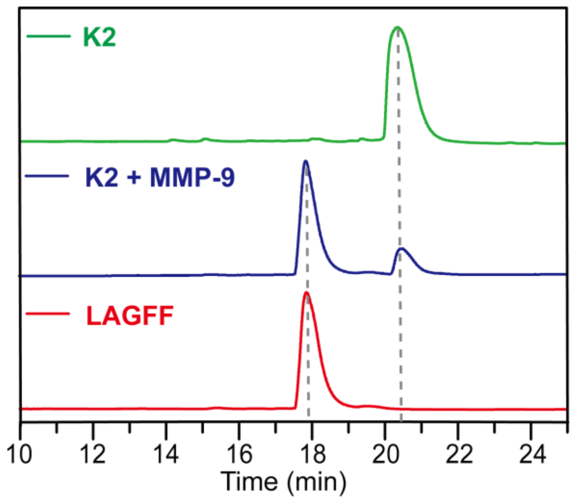

Figure S7. HPLC traces of 1mM **K2** (green) and incubated with 100 ng/mL MMP-9 (blue) (pH 7.4) for 24 h at 37 °C, and synthesized LAGFF as a control (red).

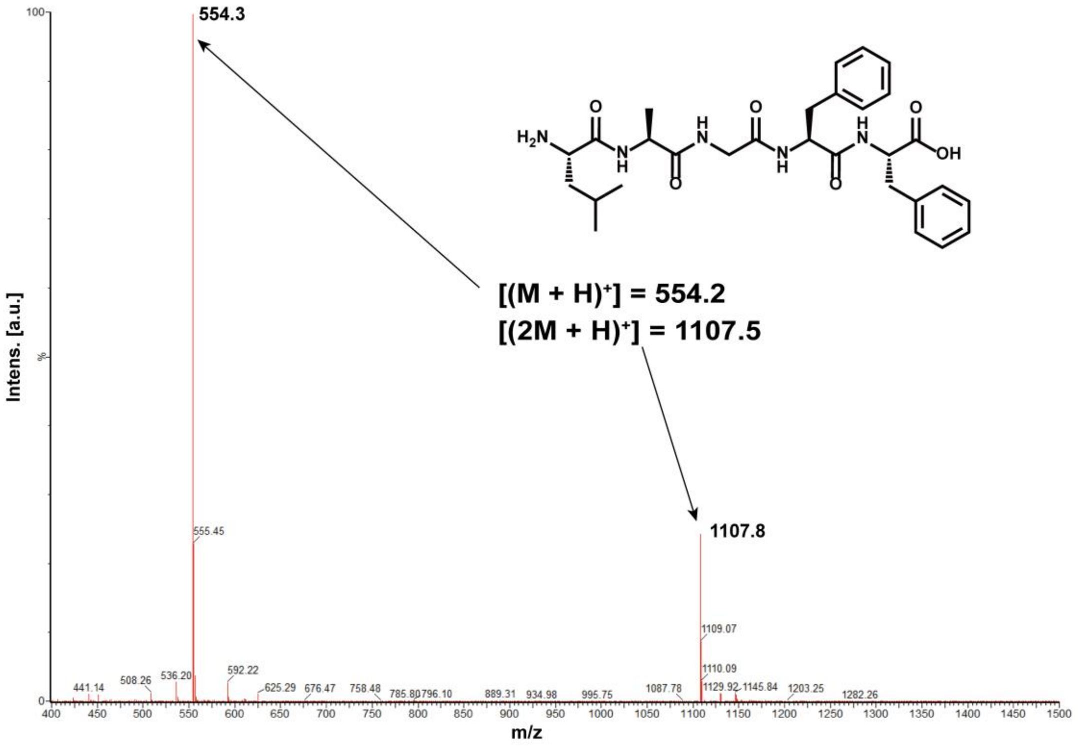

Figure S8. ESI-MS spectrum of the peak at 18 min on the HPLC trace of **K2** digestion with MMP-9 in Figure S5.

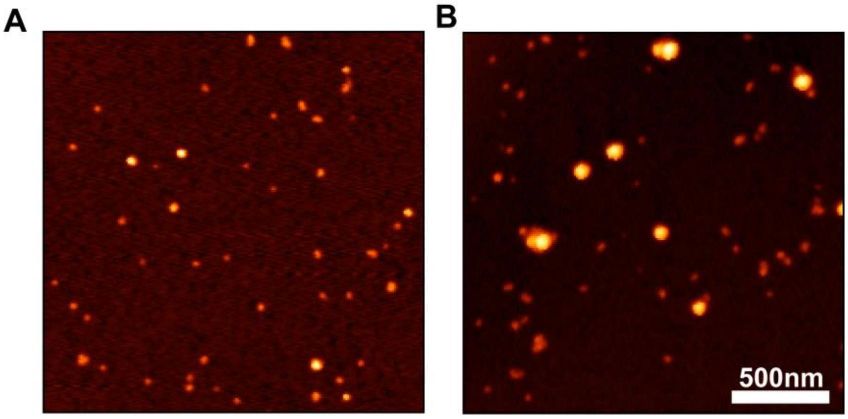

Figure S9. AFM images of **K2 micelles** (A), and **bFGF@K2 micelles**. Scale bar 500 nm.

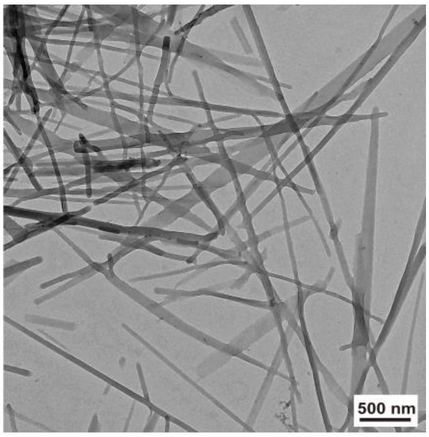

Figure S10. TEM (120 kV) image of peptide LAGFF (10 mM) after a heating-cooling process.

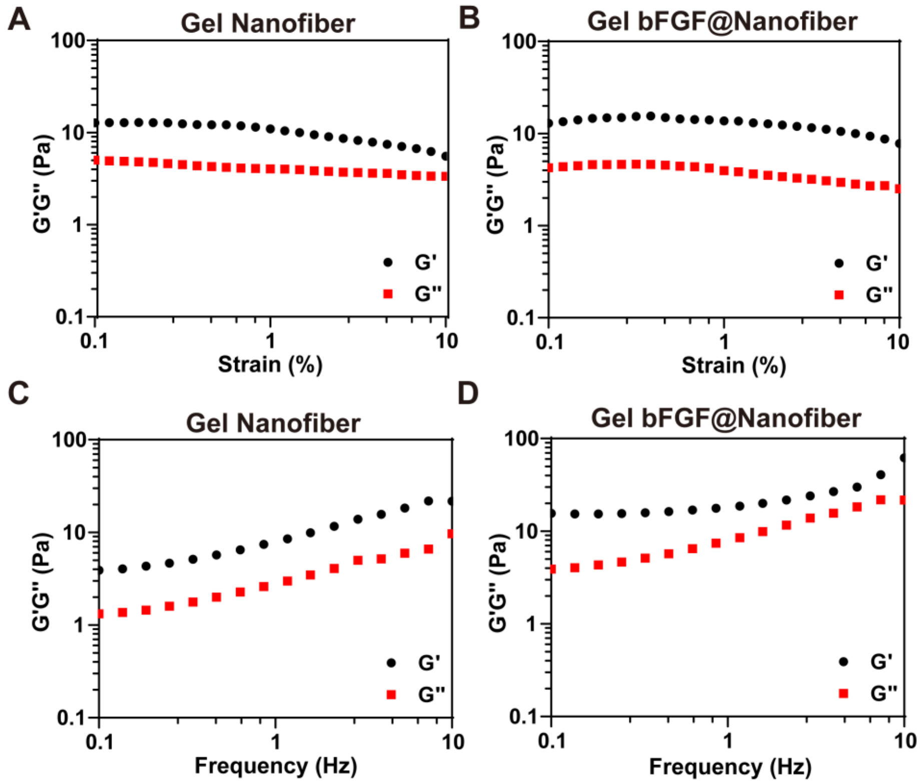

Figure S11. Strain dependence of the dynamic storage moduli (G') and the loss moduli (G'') of **nanofibers** hydrogel (A) and **bFGF@Nanofibers** hydrogel (B) at the frequency of 1 Hz. Storage modulus (G') and loss modulus (G'') values of **nanofibers** hydrogel (C) and **bFGF@Nanofibers** hydrogel (D) at different frequencies, a strain of 0.1%, condition: pH 7.4, 25 ℃.

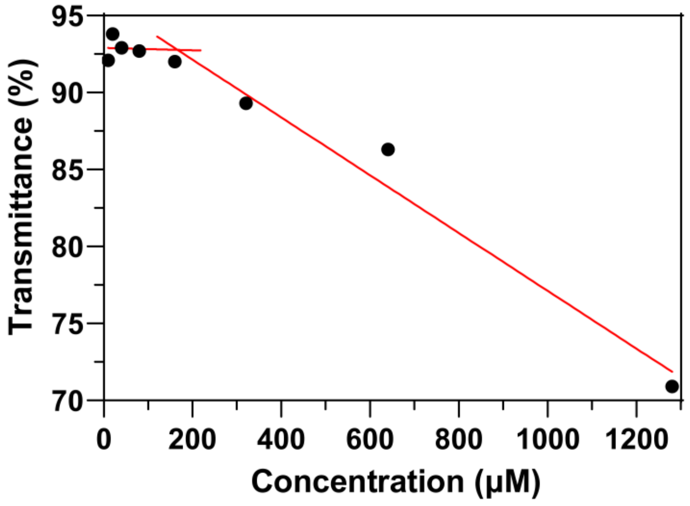

Figure S12. Gelator concentration-dependent transmittance at 600 nm of dilutions of Gel-LAGFF.

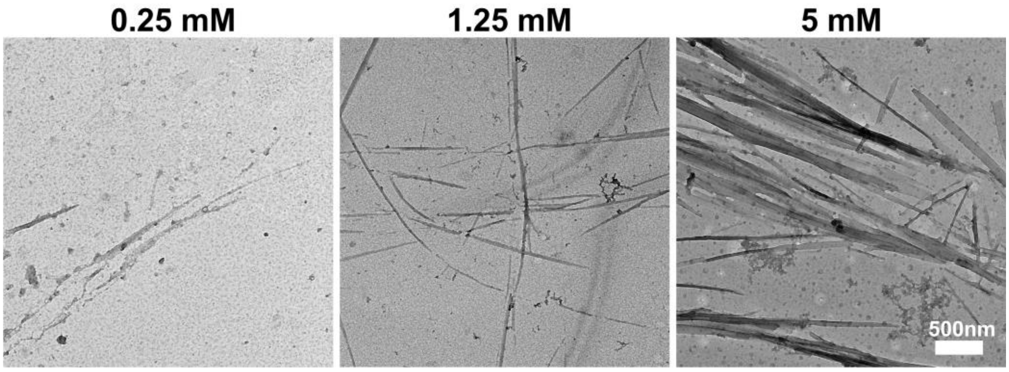

Figure S13. TEM images of 0.25mM, 1.25mM, and 5 mM **bFGF@K2 micelles** incubated with 100 ng/mL MMP-9 for 24 h.

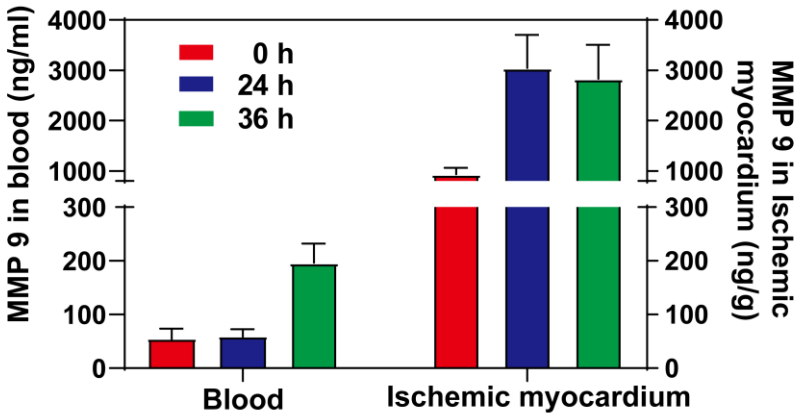

Figure S14. Quantitative results of expression levels of MMP-9 in the blood and ischemic myocardium of rats 24 h and 36 h post MI/R; n = 3 for each time point.

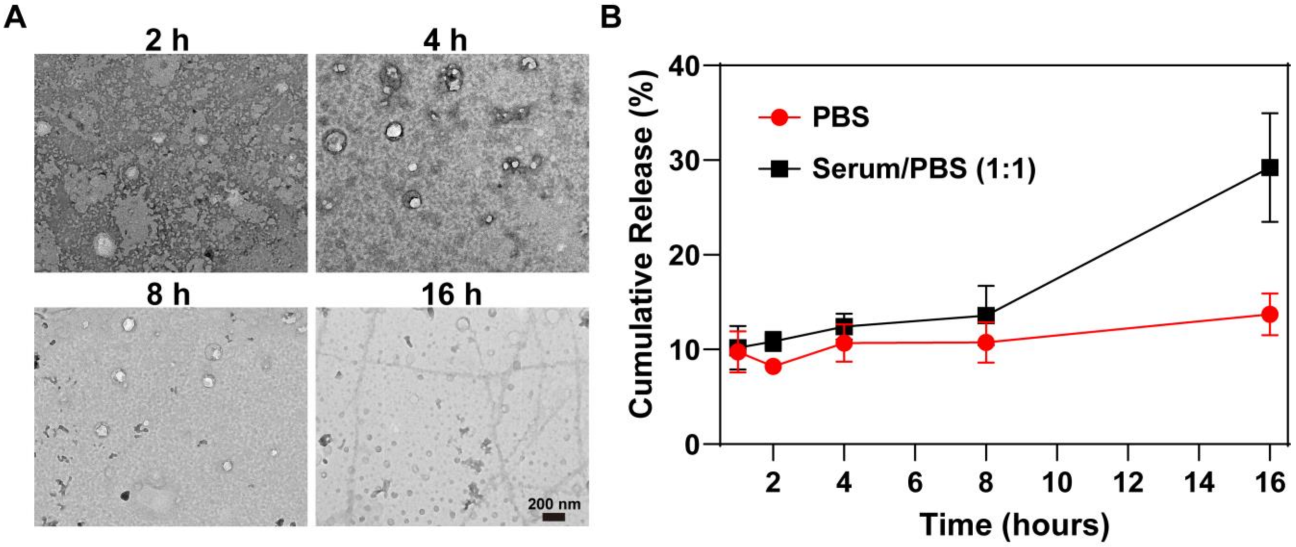

Figure S15. (A) TEM images of **bFGF@K2 micelles** after different incubation times in serum/PBS (1:1) solution. Serum was obtained from the rats at 36 h post-MI/R. (B) Cumulative release of **bFGF** from **bFGF@K2 micelles** in different incubation solutions.

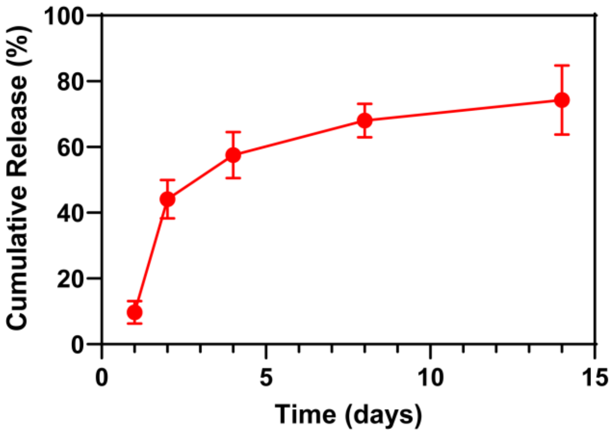

Figure S16. In vitro cumulative release of **bFGF** from **bFGF@Nanofiber** hydrogel in PBS (0.01 M, pH 7.4) at 37 ℃.

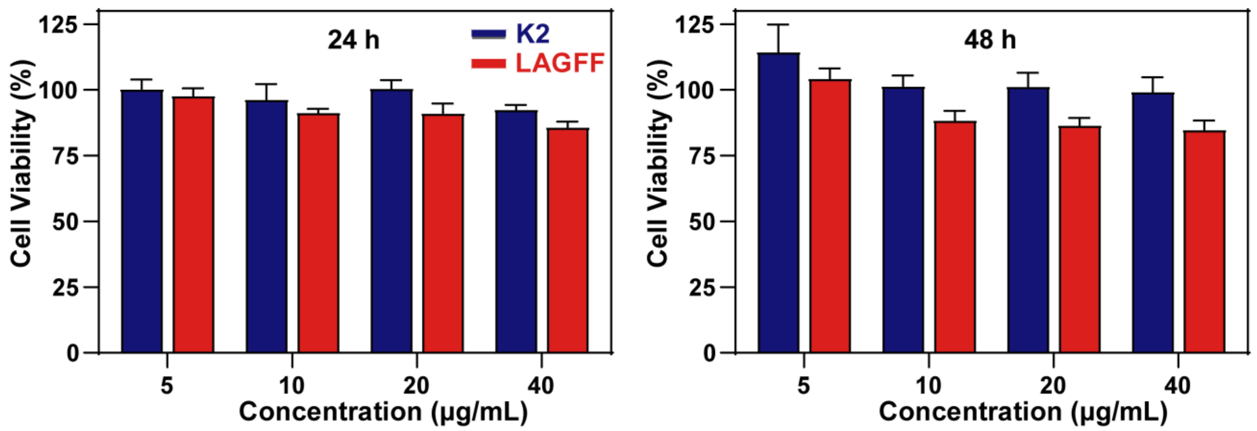

Figure S17. MTT assays of peptide **K2** and LAGFF on H2C9 cells after 24 h and 48 h incubation.

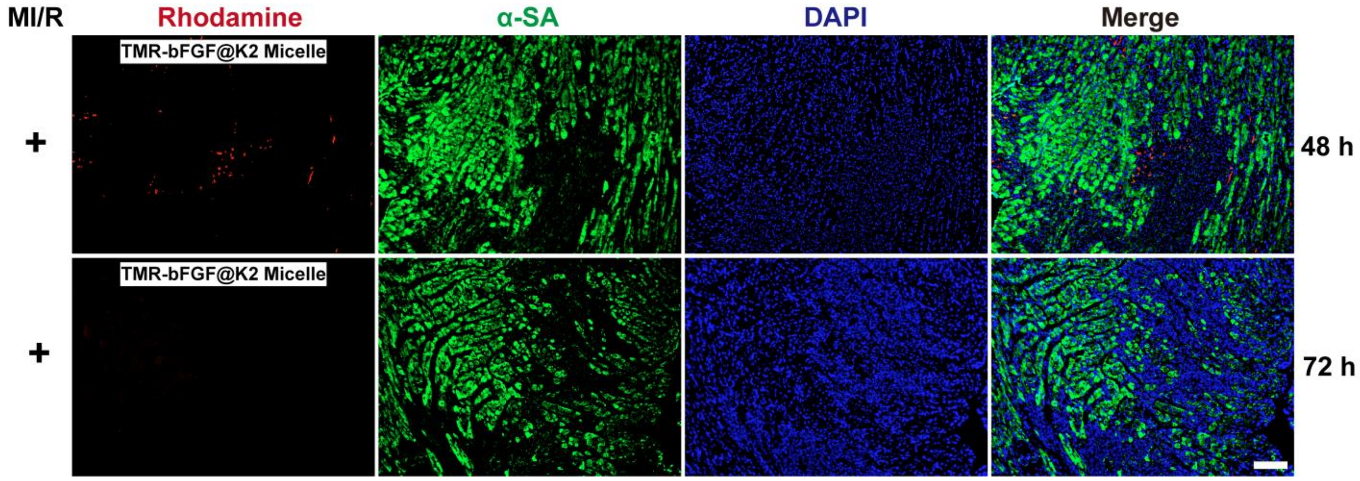

Figure S18. Fluorescent images of ischemic myocardial tissue of MI/R rats at 48 h (top lane) or 72 h (bottom lane) post tail vein injection of **TMR-bFGF@K2 micelle**. **bFGF** was labeled with **TMR** (red), myocardium was labeled with α-SA (green) and nuclei were stained with DAPI (blue). Scale bar: 100 μm.

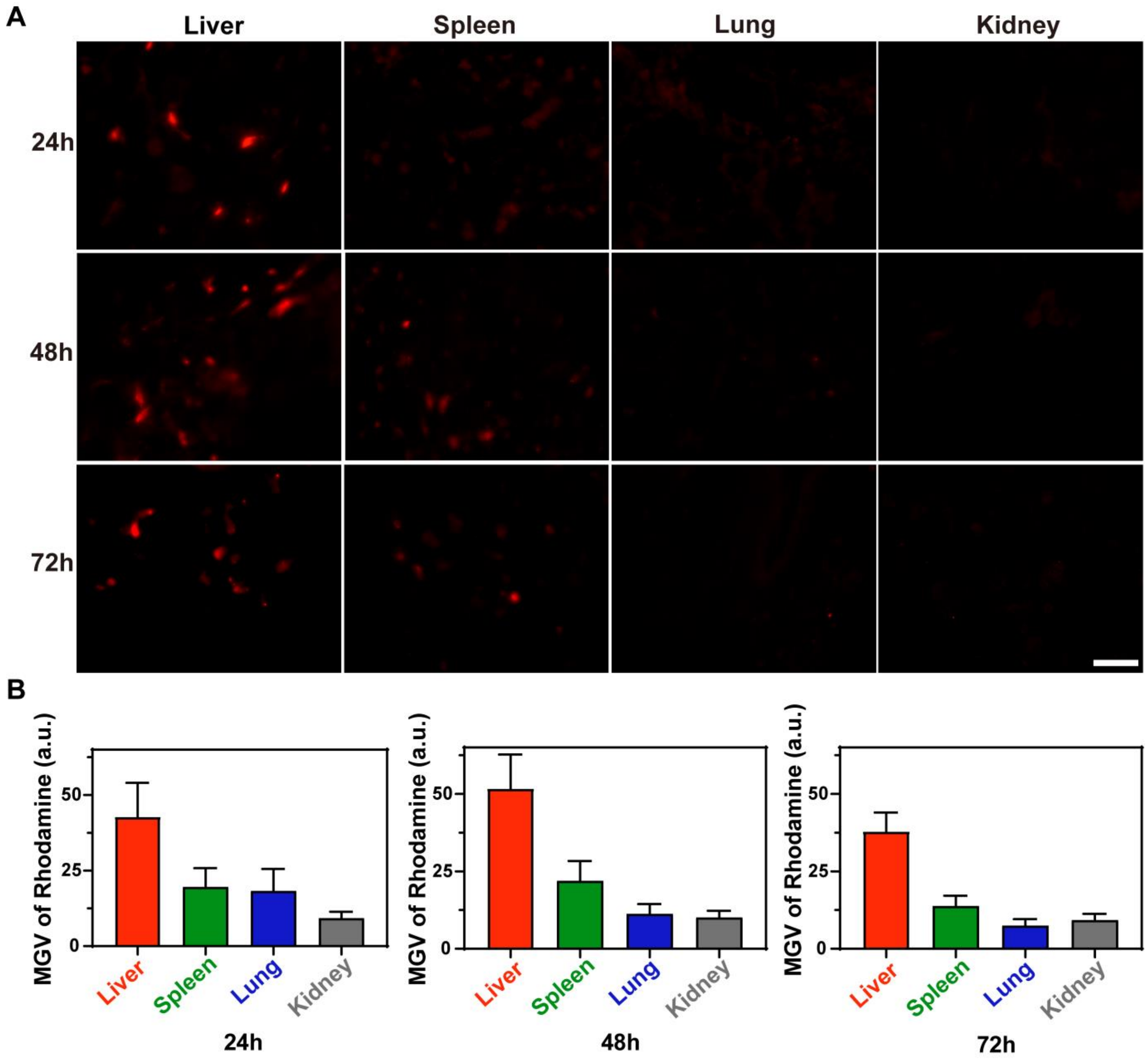

Figure S19. (A) Fluorescent images of major organs from MI/R rats at 24 h (top lane), 48 h (middle lane), or 72 h (bottom lane) post **TMR-bFGF@K2 micelle** tail vein injection. Scale bar: 50 μm. (B) Quantitative analysis of mean gray value (MGV) (n = 3).

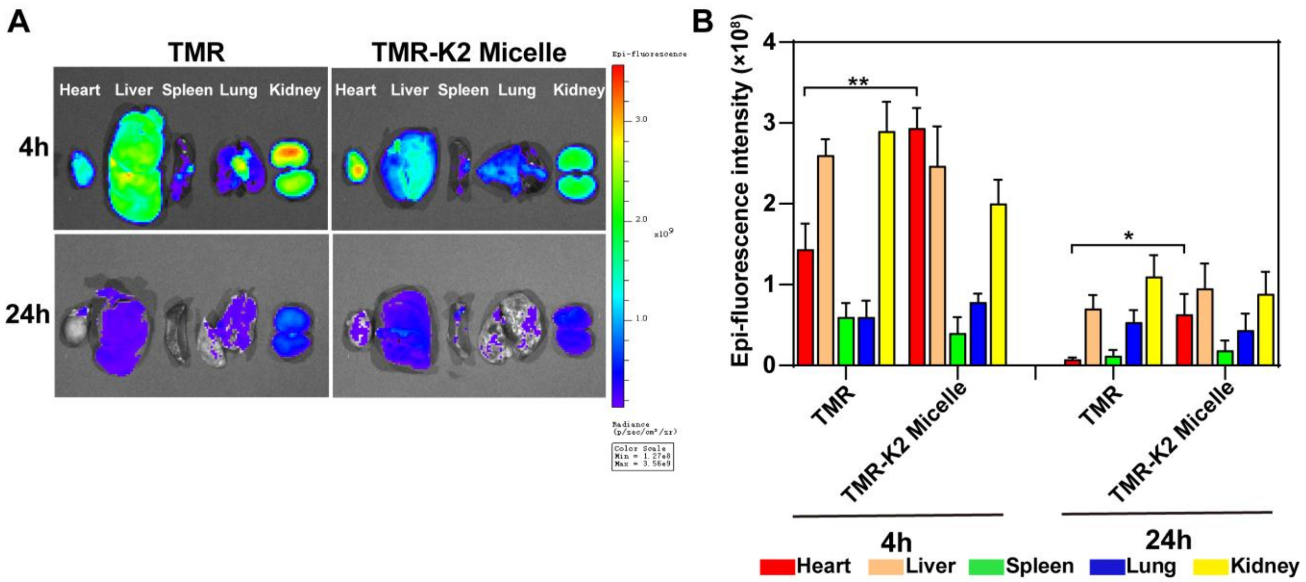

Figure S20. Biodistributions of **TMR** and **TMR-K2 micelles** after intravenous delivery in rats with MI/R injury. (A) Representative ex vivo fluorescent imaging of rat organs (heart, liver, spleen, lung, and kidney) at 4 h and 24 h post-intravenous injections of **TMR** or **TMR-K2 micelle**. (B) Quantitative analysis of fluorescent intensities in (A) (n = 3 per group).

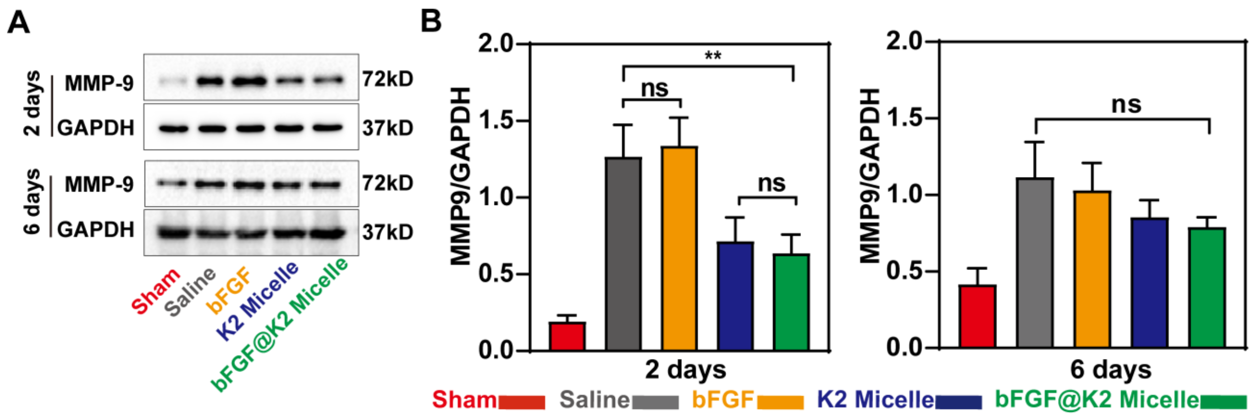

Figure S21. Western blotting (A), and quantification analysis (B) of MMP-9 level of rats in each group 2 days and 6 days post tail vein injection (n = 3). The results are presented as the mean ± SD of the MMP-9 level relative to reference GAPDH. $^{**}P < 0.01$.

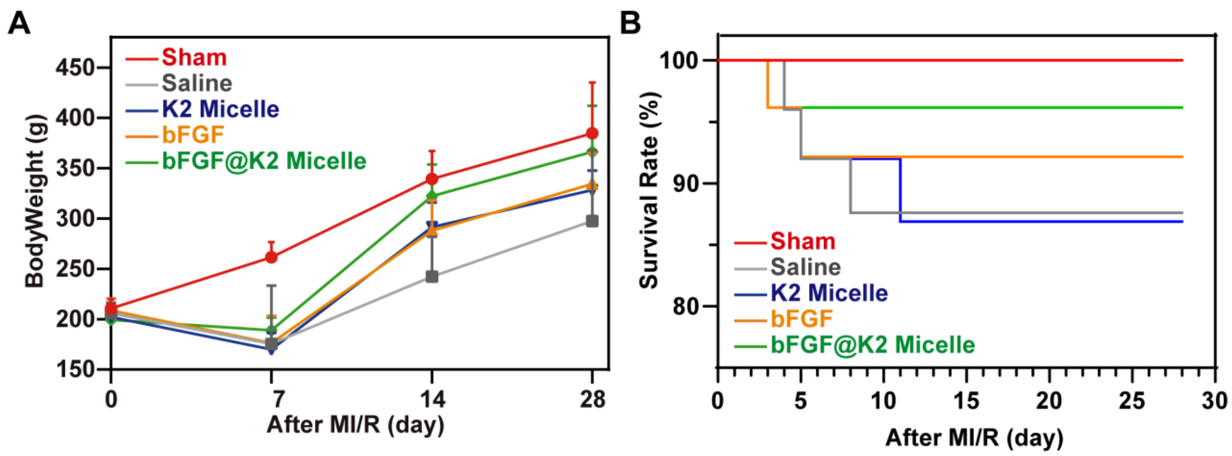

Figure S22. (A) Body weights of rats in each group after MI/R. (B) Kaplan-Meier survival curves of rats during the 28 days in each group after MI/R.

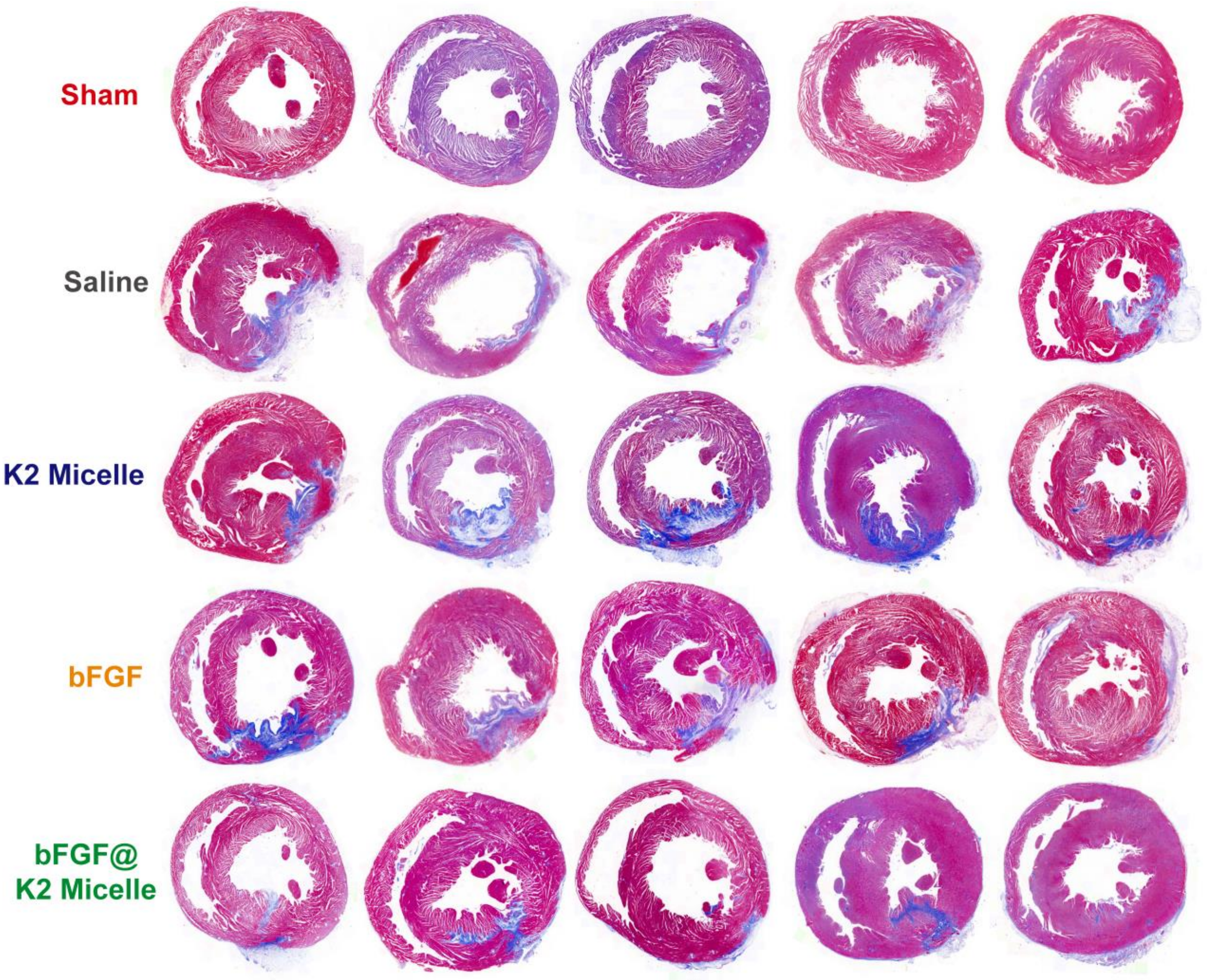

Figure S23. The optical images of Masson's trichrome stained rat heart sections in each group (n = 5).

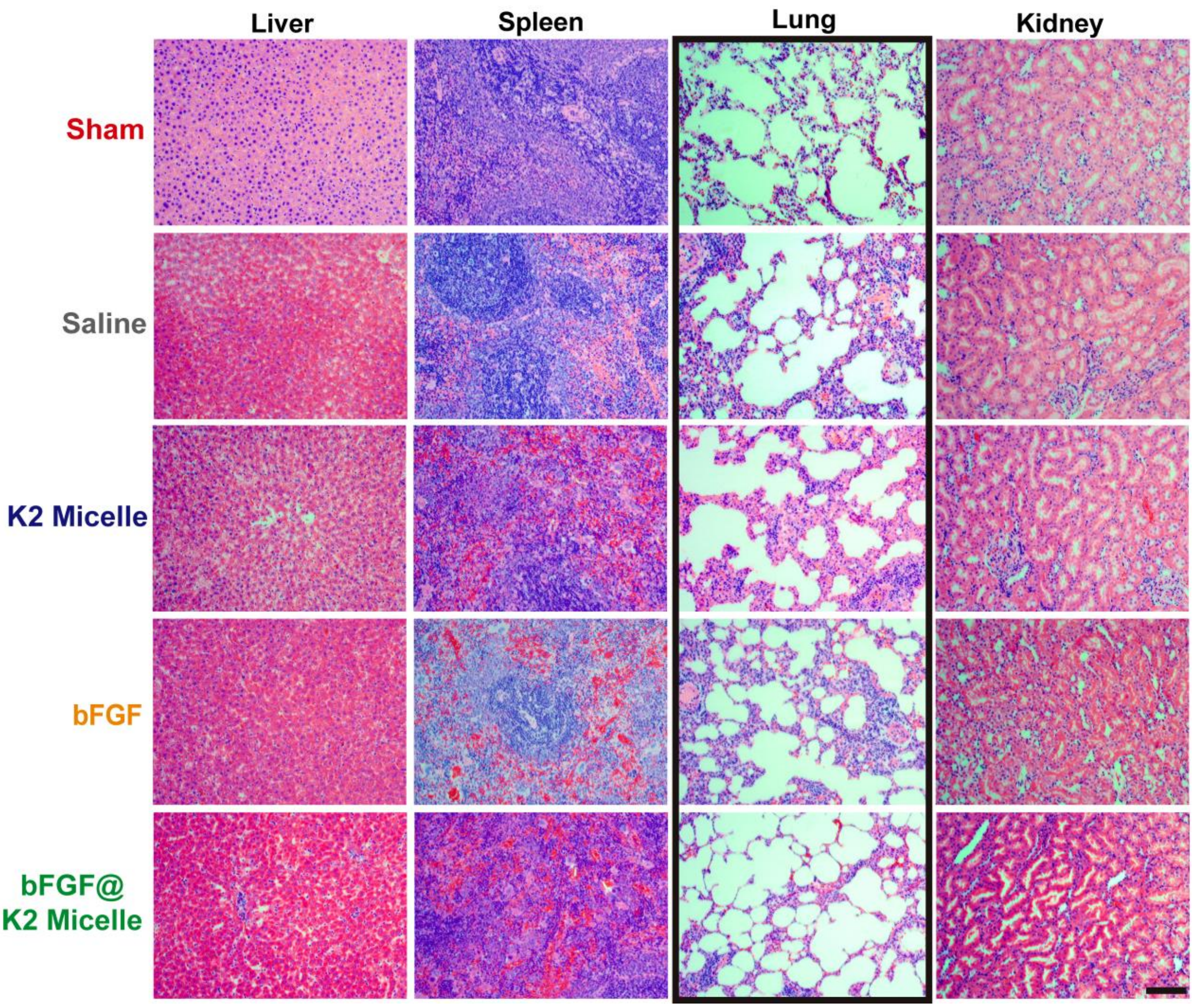

Figure S24. H&E stained images of major organs (liver, spleen, lung, kidney) harvested from five groups of rats sacrificed 28 days after MI/R. Scale bar: 50 μm.

Table S1. HPLC condition for the purification of peptide **K2**

**Time (min)**	**Flow (ml/min)**	**H_2O% (0.1%TFA)**	**CH_3OH% (0.1%TFA)**
0	12.0	80	20
3	12.0	80	20
35	12.0	20	80
37	12.0	20	80
38	12.0	80	20
40	12.0	80	20

Table S2. HPLC condition for Figure S5

**Time (min)**	**Flow (ml/min)**	**H_2O% (0.1%TFA)**	**CH_3OH% (0.1%TFA)**
0	1.0	85	15
3	1.0	85	15
35	1.0	40	60
37	1.0	40	60
38	1.0	85	15
40	1.0	85	15
